# Adopting life-cycle HTA: a tumor-agnostic precision oncology index economic evaluation from publicly available reimbursement reviews

**DOI:** 10.1017/S0266462325100111

**Published:** 2025-06-24

**Authors:** Gemma Cupples, Emanuel Krebs, Deirdre Weymann, Cheryl Ho, Dean A. Regier

**Affiliations:** 1 Regulatory Science Lab, BC Cancer Research Institute, Vancouver, BC, Canada; 2 Faculty of Health Sciences, Simon Fraser University, Vancouver, BC, Canada; 3 Department of Medical Oncology, BC Cancer, Vancouver, BC, Canada; 4 Department of Medicine, Faculty of Medicine, University of British Columbia, Vancouver, BC, Canada; 5 School of Population and Public Health, Faculty of Medicine, University of British Columbia, Vancouver, BC, Canada

**Keywords:** health technology assessment, life-cycle HTA, economic evaluation, precision oncology, tumor-agnostic therapies

## Abstract

**Objectives:**

Life-cycle health technology assessment (HTA) requires an index economic model to establish how estimated cost-effectiveness evolves with emerging evidence. We developed an open-source index economic evaluation of entrectinib, a tumor-agnostic therapy with conditional market authorization. Our objective was to replicate the initial HTA report from publicly available information, aiming to identify key operational and methodological aspects for operationalizing life-cycle decision-making.

**Methods:**

We used partitioned survival analysis to determine tumor-agnostic and tumor-specific cost-effectiveness, using publicly available HTA reviews for parameterization. We estimated incremental costs in 2021 Canadian and US dollars (CAD and USD) from a public-payer healthcare perspective, quality-adjusted life years (QALYs), and incremental net monetary benefit (INMB). We assessed the impact of treatment effectiveness, extrapolation assumptions, and next-generation sequencing (NGS) costs.

**Results:**

One-third of the parameters (*n* = 30) were unavailable in the Canadian reimbursement review and were sourced from international reviews. Tumor-agnostic incremental costs were CAD 68,451 (95 percent confidence interval: 35,466, 92,155) and USD 54,608 (28,294, 73,518), and QALYs were 0.13 (−0.42, 0.42), yielding INMB CAD −55,803 at 100,000/QALY (USD −44,518). Full extrapolation of treatment effectiveness also yielded negative INMB (CAD −66,664). Inclusion of NGS costs diminished the expected value. Heterogeneity was considerable across tumor indications.

**Conclusions:**

We developed an open-source index economic evaluation to operationalize life-cycle HTA for a conditionally authorized tumor-agnostic therapy. Our findings outline key operational and methodological considerations necessary for the development of index economic models that support life-cycle HTA, offering insights into their potential integration into regular HTA and policy decision-making processes.

## Introduction

Decision-makers rely on health technology assessment (HTA) evidence when deliberating on the reimbursement of health products. The HTA package typically includes regulatory evidence on safety and efficacy alongside evidence of patient value, cost-effectiveness, and budget impact (1). Reimbursement deliberations should be informed by processes that are transparent (2) and allow flexibility for generating jurisdiction-specific evidence (3). Any uncertainties in the available evidence should be made accessible for decision-maker consideration (4).

For an overwhelming majority of recent precision oncology trials, the absence of control groups or randomization has resulted in substantial evidentiary uncertainty (5). Advancements in genomics have improved our ability to differentiate cancers based on their genetic alterations, and emerging targeted therapies treat genetic biomarkers irrespective of cancer site (6). These tumor-agnostic oncology products, implemented across multiple cancer types in small patient groups, are frequently evaluated in single-arm clinical trials that lack a standard of care counterfactual (7;8). Uncertainty in the causal relationship between intervention and outcome impedes our understanding of safety and efficacy, patient value, and cost-effectiveness (7), challenging HTA deliberations. As a result, more treatments are gaining approval under conditional approval mechanisms that allow for reduced evidentiary requirements, contingent on further evidence development (9). Life-cycle HTA is positioned to address these requirements for additional evidence generation by iteratively assessing clinical, economic, and societal impacts of technologies across their life cycle, from development to de-adoption (7;10;11). We define life-cycle HTA as the standardization of data generation and collection, combined with methods to produce decision-grade evidence for deliberation at each phase of a technology’s life cycle (7;8).

Adopting a life-cycle framework for cost-effectiveness analysis informs dynamic value for money and acceptable pricing, providing insight into the importance of collecting additional data to augment the current evidence base. The economic models used within this framework are sequentially updated, using real-world evidence, to guide deliberations on the appraisal, re-appraisal, and de-adoption of technologies (7). Currently, cost-effectiveness models submitted to HTA bodies take a static approach to on/off decision-making and are rarely updated when new evidence emerges (12–15). Transparency, flexibility, and consistency of evaluative frameworks are important elements that can facilitate the adoption of life-cycle HTA (11). Taking a more holistic approach to the conduct of life-cycle economic evaluation (as opposed to *de novo* modeling for generating context- and jurisdiction-specific economic evidence) can promote robust, fit-for-purpose evidence generation and enhance the efficiency of deliberative processes by assisting HTA bodies facing increasing demands and important resource constraints.

Life-cycle HTA requires an index economic model to establish how estimated cost-effectiveness evolves as new evidence emerges. Economic evaluations from HTA reimbursement reviews provide the first assessment of a new technology’s value. We propose utilizing publicly available information from these reviews to establish an index model, aiming to identify key operational and methodological aspects required for index economic modeling to support life-cycle HTA. We present a case study for entrectinib (Rozlytrek), a tumor-agnostic therapy with conditional market authorization, replicating the economic evaluation submitted to Canada’s Drug Agency (CDA-AMC) (16–18). To promote stakeholder engagement, information sharing, and transparency, we provide open-source code to serve as a replicable and modifiable benchmark, which can be adapted with jurisdiction-specific parameter inputs to guide localized reimbursement decisions.

## Methods

Our analysis used publicly available data from reimbursement reviews to reproduce the cost-effectiveness results from the CDA-AMC’s economic evaluation of entrectinib. Throughout, we identify challenges in the operationalization of this index economic evaluation to support life-cycle HTA.

### Setting

Entrectinib is a novel, tumor-agnostic therapy targeting cancers expressing neurotrophic tyrosine receptor kinase (NTRK+) gene fusion. In Canada, the therapy received conditional market authorization for the treatment of patients with advanced or metastatic extracranial solid tumors with no satisfactory treatment options, based on promising evidence, with the condition that the drug manufacturer would conduct post-market confirmatory trials for clinical benefit (17). Entrectinib then received a recommendation for conditional reimbursement from the CDA-AMC (16). The HTA review highlighted quality concerns relating to model structure, tumor-agnostic analyses, comparator definitions, tumor weighting, and exclusion of treatment waning effects (16). CDA-AMC addressed these issues where possible, but limitations in data availability constrained the possible revisions.

### Intervention and comparator

The intervention was treatment with entrectinib for NTRK+ patients with any advanced cancer diagnosis (16). The CDA-AMC reimbursement review considered outcomes data for 121 intervention patients who enrolled in one of three basket trials (ALKA-372-001, STARTRK-1, and STARTRK-2) and initiated entrectinib treatment between March 2012 and July 2019 (6;16). The comparator was standard care for treating advanced cancer in patients with unknown NTRK fusion status, defined using a combination of published estimates for established therapies for each tumor indication represented in the basket trials.

The intervention and comparator groups included multiple tumor indications: breast, colorectal, non-small cell lung (NSCLC), pancreatic, thyroid, sarcoma, neuroendocrine, and mammary analogue secretory carcinoma (MASC). A category for “other” tumor indications, comprising lower-prevalence cancer types based on trial population, included cholangiocarcinoma, endometrial, head and neck, neuroblastoma, ovarian, and cancer of unknown primary. We captured variation in the clinical presentation of this rare variant via prevalence estimations informed by the CDA-AMC reimbursement review, calculated from annual cancer prevalence by type in Canada outside of Quebec, the proportion diagnosed at Stage III/IV, and the prevalence of NTRK fusions per tumor.

### Analysis framework

Our analysis is built on a partitioned survival analysis (PartSA) framework (Supplemental Figure S1) (19;20), incorporating revisions from the CDA-AMC reimbursement review for entrectinib (16). PartSA consists of three health states: progression-free, progression, and death. Parameterization of survival curves for overall survival (OS) and progression-free survival (PFS) determines the proportion of patients in each health state; progression-free is estimated from the area under the PFS curve, while disease progression is the difference between OS and PFS. Given the tumor-agnostic indication for entrectinib, we stratified analyses by cancer type, with tumor-agnostic results estimated from a prevalence-weighted average of each tumor indication. Additional details are provided in the Supplementary File (Section 1).

All code is publicly available, along with a dashboard to enable adaptation of selected input parameters for jurisdiction-specific analyses, at https://regulatory-science-lab.github.io/.

### Inputs

Where possible, we prioritized the model parameters detailed in the CDA-AMC reimbursement review. For missing inputs, we used the National Institute for Health and Care Excellence (NICE) reimbursement review. All parameters were obtained from these sources unless otherwise stated. Additional details are provided in the Supplementary File (Section 1). Table 1 details the tumor-agnostic model parameters, and tumor-specific parameters are summarized in Supplementary Tables 1–3.

**Table 1.** Input parameters

Parameter name	Value	PSA	Source
Time horizon	10 years	–	CDA-AMC
Cycle length	7 days	–	CDA-AMC
Cost discount rate	1.5%	–	CDA-AMC
Utility discount rate	1.5%	–	CDA-AMC
Prevalence	Available in Supplemental Table 1	Dirichlet^a^	CDA-AMC
Number needed to screen with NGS to find 1 NTRK fusion	Available in Supplemental Table 3	–	CDA-AMC
Survival data			
OS, entrectinib	Available in Supplemental Table 1	Truncated normal or Beta	CDA-AMC
PFS, entrectinib	Available in Supplemental Table 1	Truncated normal or Beta	CDA-AMC
OS, comparator	Available in Supplemental Table 1	Truncated normal	NICE
PFS, comparator	Available in Supplemental Table 1	Truncated normal	NICE
Costs, CAD^b^ (weekly unless stated)			
Entrectinib drug	2,002 (USD 1,597.13)	–	CDA-AMC
Entrectinib administration	0	–	CDA-AMC
Comparator drug	Available in Supplemental Table 2	–	CDA-AMC
Comparator administration	Available in Supplemental Table 2	±20%^a^	NICE
Care, PFS, entrectinib	104.86 (USD 83.65)	±20%^a^	NICE
Care, PFS, comparator	133.49 (USD 106.49)	±20%^a^	NICE
Care, progression	139.42 (USD 111.22)	±20%^a^	NICE
Adverse events (single)	1,494.01 (USD 1,191.87)	±20%^a^	NICE
NGS (single)	1,400 (USD 1,116.87)	–	CDA-AMC
NTRK testing, comparator (single)	0	–	CDA-AMC
NTRK testing, entrectinib (single)	Available in Supplemental Table 3	±20%^a^	CDA-AMC
Non-cancer care	Available in Supplemental Table 2	±20%^a^	CDA-AMC
Health state utilities (per state)			
Progression-free	0.788	±10%^a^	CDA-AMC
Progressive disease	0.642	±10%^a^	CDA-AMC

a Distributions were not provided in HTA documents and thus assumed by the authors.

b Costs in 2021 Canadian dollars. CDA-AMC, Canada’s Drug Agency; NICE, National Institute for Health and Care Excellence; NGS, next-generation sequencing; NTRK, neurotrophic tyrosine receptor kinase; OS, overall survival; PFS, progression-free survival; PSA, probabilistic sensitivity analysis.

#### Survival data

Stratified entrectinib survival data were mostly available in the form of median OS and PFS estimates. Where median survival was not achieved during the trial, we instead used a critical point on the survival curve, where 50 percent of the population remained at risk (12). For tumor types with five or fewer patients in the trial population, we assumed only incremental costs of treatment and no incremental quality-adjusted life years (QALYs).

Information to characterize the comparator arm was not provided in the CDA-AMC reimbursement review. To supplement the missing evidence, we used data from NICE. Median survival was estimated by averaging the median OS and PFS outcomes for several previously NICE-recommended therapies per tumor indication. Individual therapies that comprised the standard care arm were not reported in the NICE reimbursement review, preventing comparison with clinical practice in Canada. To estimate survival curves for the analysis, we fitted exponential curves through the median OS and PFS data for both arms in the analysis, in line with CDA-AMC and NICE reimbursement reviews.

#### Utility weights

We assumed that the health-state utilities were tumor agnostic and equivalent between the entrectinib and comparator arms, in alignment with the CDA-AMC reimbursement review. The sources underlying utility values were not provided.

#### Resource use and costs

Tumor-agnostic costs consisted of care, adverse events, and entrectinib treatment and administration. Non-cancer care and standard-of-care treatment costs were tumor-specific. To estimate the costs for the tumor indication “other,” we used a weighted average of the costs for all other tumor indications. We assumed that patients received entrectinib treatment up until the median time to treatment discontinuation, incorporated through time-dependent costs. All costs were expressed in 2021 Canadian dollars (CAD) and US dollars (USD). Canadian costs were converted to USD using the Bank of Canada’s historical exchange rates (21). To ensure equivalence between jurisdictions, we compared the entrectinib list price included in both NICE and CDA-AMC reimbursement reviews. The adjusted cost estimates do not impact the cost-effectiveness decision. Additional details are provided in the Supplementary File (Section 1.4).

### Model validation

We completed a checklist developed by CDA-AMC (22), which aims to validate the accuracy and relevance of economic evaluations that inform decision-making.

### Cost-effectiveness analysis

#### Base case

We report both tumor-agnostic and tumor-specific incremental costs, QALYs, and incremental cost-effectiveness ratios (ICERs). We used cost and effectiveness outcomes to calculate the incremental net monetary benefit (INMB) at willingness-to-pay (WTP) thresholds of CAD 50,000/QALY and CAD 100,000/QALY. The study was conducted from the Canadian healthcare payer’s perspective, with a discount rate of 1.5 percent for both costs and utilities. We used a weekly cycle length over a time horizon of 10 years.

Curves fit to the median survival data for the comparator were extrapolated to the model time horizon. The efficacy of entrectinib beyond the trial is highly uncertain, due to a lack of evidence supporting long-term outcomes. Following best practice recommendations, we restricted the efficacy of entrectinib to mitigate overestimation of the incremental benefit (1;12;14). This approach is consistent with the CDA-AMC reimbursement review. Additional details are provided in the Supplementary File (Section 1).

We conducted a probabilistic analysis to assess the impact of parameter uncertainty on our results, using 1,000 iterations while sampling all parameters simultaneously. Detailed information regarding the distributions used to characterize parameter uncertainty is provided in the Supplementary File (Section 2, Tables S1 and S2).

#### Survival extrapolation analysis

**Figure 1.**
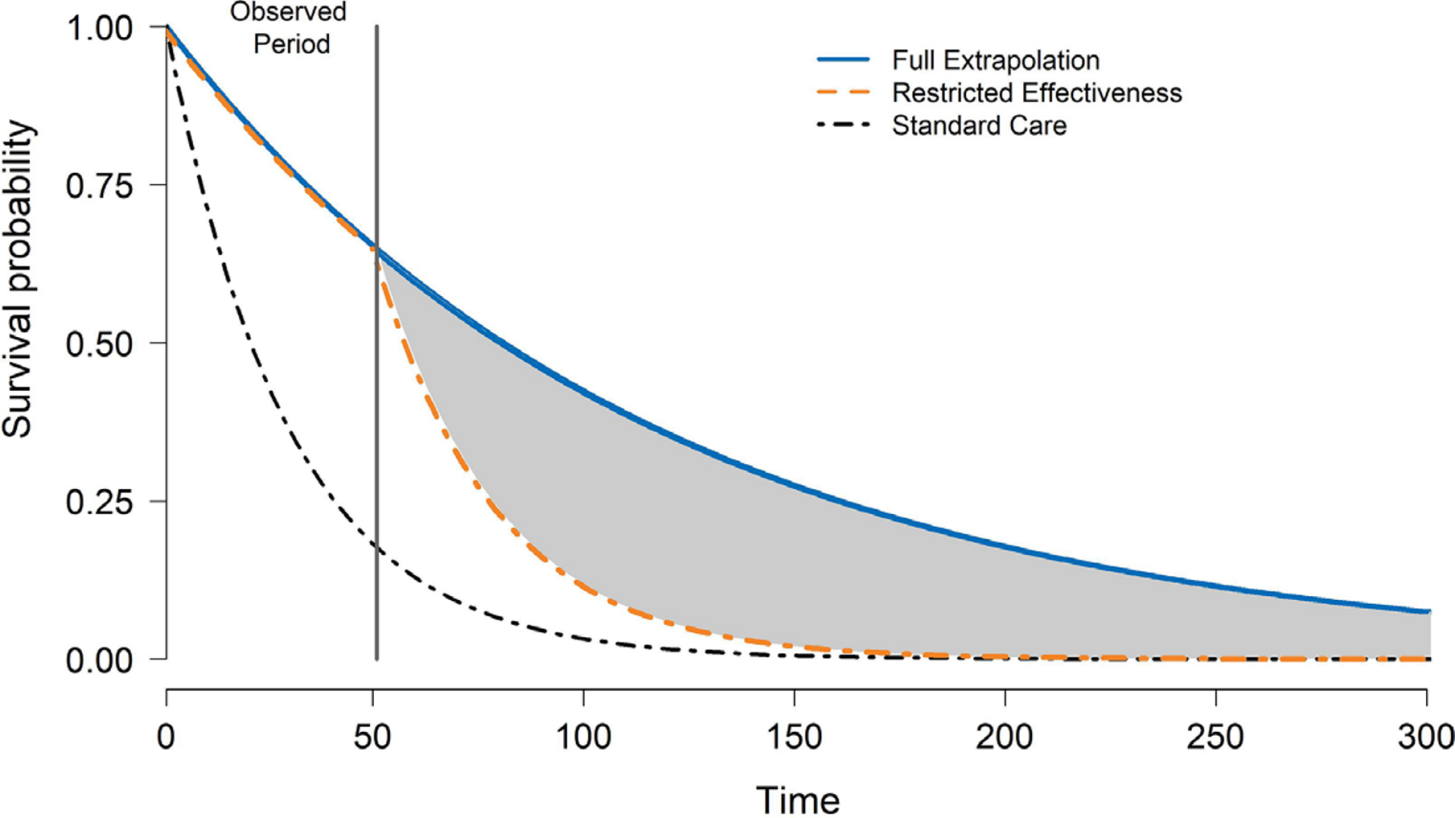
Illustration of conservative and optimistic assumptions for the extrapolation of hypothetical treatment effectiveness on overall survival.

Our base case represents a conservative scenario for long-term entrectinib effectiveness. We conducted a scenario analysis by extrapolating the effectiveness of entrectinib to the model time horizon (Figure 1). This optimistic approach is in line with the initial sponsor submission (16).

#### Testing scenario analysis

To capture the diagnostic testing pathway and associated costs, we consider a scenario analysis to assess the impact of next-generation sequencing (NGS) panel costs that are recommended to determine eligibility to receive entrectinib based on NTRK fusion status. Testing was included for all tumor indications, in line with the CDA-AMC reimbursement review. The use of NGS was based on clinician preference for a test that can screen for multiple gene variants (16). We estimated testing costs for the entrectinib arm from the number needed to screen to identify one NTRK-positive patient, determined from the per-tumor prevalence of NTRK gene fusions (Supplemental Table 3). The NGS panel unit costs were taken from the CDA-AMC reimbursement review. Testing costs were added to the first-time cycle in the model, as testing occurs before treatment initiation. We assumed that the comparator arm was untested, since NTRK status is unknown. Reimbursement for NGS testing will vary by jurisdiction and tumor indication; as such, we include the flexibility to adjust these costs in the code and accompanying dashboard to account for jurisdiction-specific funding arrangements.

#### Decision algorithm

We present cost-effectiveness frontiers for the deterministic analysis of the base case and testing scenario. The frontier considers the cost-effectiveness of every combination of tumor indications to inform funding decisions for a tumor-agnostic therapy that can be updated as new evidence is generated throughout its life cycle.

#### Value of information

Value of information analyses can help identify parameters that drive uncertainty in cost-effectiveness outcomes and determine the potential societal benefit of continued research to reduce these uncertainties. We calculated the tumor-agnostic and tumor-specific expected value of perfect information (EVPI), which represents the monetary value to healthcare decision-makers of removing all uncertainty from the cost-effectiveness analysis. We evaluate both the base case and the optimistic extrapolation scenario.

## Results

Throughout our analysis, we identified constraints related to parameter availability, the characterization of the comparator arm, and the analysis framework, all of which impeded the operationalization of this index economic evaluation. Across model input parameters, 34 percent (*n* = 30) were unavailable in the CDA-AMC reimbursement review, notably standard-of-care survival data used to construct the comparator arm. In addition, parameters of the distributions for probabilistic analysis were only available for entrectinib survival estimates.

The CDA-AMC checklist (Supplemental Table 4) highlighted several issues in the economic analysis, including a lack of adverse event reporting and model transparency, validity, and representativeness. Our analysis performed well across code scrutiny, emphasizing the accuracy and robustness of our code.

### Base case

**Figure 2.**
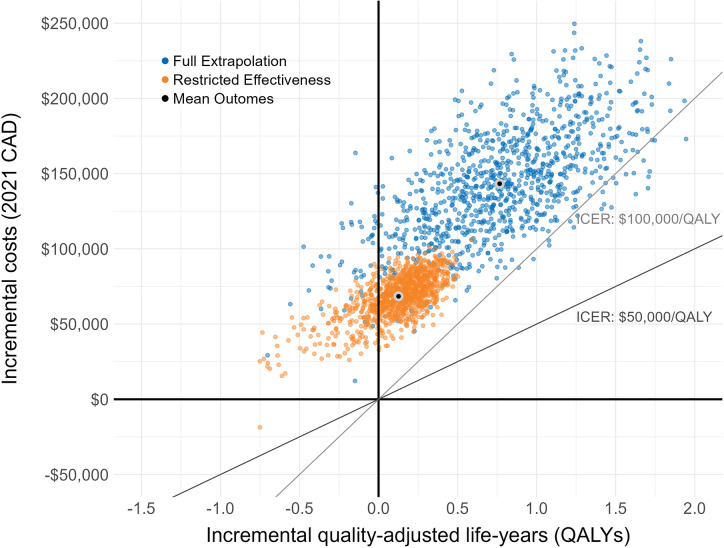
Cost-effectiveness plane from 1,000 simulations comparing entrectinib to tumor-specific standard of care, without NGS costs. Separate outcomes for each tumor indication were weighted using cancer prevalence estimates. CAD, Canadian dollars; ICER, incremental cost-effectiveness ratio; NGS, next-generation sequencing; PSA, probabilistic sensitivity analysis.

Base case tumor-agnostic and tumor-specific incremental outcomes are provided in Table 2, with USD results in Supplemental Table 5. Cost-effectiveness planes are provided for the tumor-agnostic (Figure 2) and tumor-specific (Supplemental Figure S2) results.

**Table 2.** Mean tumor-agnostic and tumor-specific probabilistic cost-effectiveness outputs with restricted effectiveness of entrectinib

	Incremental cost, CAD^a^, Mean (95% CI)	Incremental QALYs, Mean (95% CI)	ICER^a^, CAD/QALY, Mean (95% CI)	% Dominated	INMB at CAD 50,000/QALY, CAD^a^, Mean (95% CI)	% Cost-effective at CAD 50,000/QALY^a^	INMB at CAD 100,000/QALY, CAD^a^, Mean (95% CI)	% Cost-effective at CAD 100,000/QALY^a^
Tumor-agnostic	68,451 (35,466, 92,155)	0.126 (−0.419, 0.421)	866,268 (180,430, 4,571,068)	20.5	−62,127 (−81,677, −40,698)	0.1	−55,803 (−89,102, −29,736)	0.1
MASC	204,501 (169,746, 251,046)	1.125 (0.634, 1.432)	191,364 (137,491, 319,500)	0	−148,264 (−195,029, −113,397)	0	−92,027 (−149,512, −50,023)	0
NSCLC	72,476 (−9,172, 124,234)	0.494 (−0.134, 0.873)	190,958 (2,626, 710,854)	3.7	−47,773 (−87,696, 16,360)	4.9	−23,071 (−74,028, 36,807)	16.1
Breast	86,134 (−13,330, 124,485)	0.173 (−1.12, 0.578)	1,286,294 (22,994, 2,511,513)	16.0	−77,504 (−106,563, −30,411)	3.7	−68,874 (−118,010, −39,391)	3.7
Colorectal	51,439 (2,604, 78,160)	0.037 (−0.437, 0.224)	1,476,479 (59,160, 5,209,581)	23.3	−49,572 (−75,150, −19,357)	1.9	−47,706 (−80,393, −17,057)	2.2
Sarcoma	78,333 (9,076, 113,659)	−0.008 (−1.064, 0.488)	Dominated	38.2	−78,727 (−110,429, −44,980)	1.9	−79,121 (−134,892, −38,335)	1.9
Thyroid	60,610 (−61,629, 110,841)	−0.357 (−2.592, 0.591)	Dominated	53.5	−78,446 (−119,370, −24,353)	10.1	−96,282 (−204,966, −30,049)	10.1
Pancreatic	23,154 (1,662, 63,438)	0 (0, 0)	Dominated	N/A	−23,154 (−63,438, −1,662)	N/A	−23,154 (−63,438, −16,62)	N/A
Neuro-endocrine	138,896 (41,784, 273,467)	0 (0, 0)	Dominated	N/A	−138,896 (−273,467, −41,784)	N/A	−138,896 (−273,467, −41,784)	N/A
Other	45,206 (8,829, 78,831)	0 (0, 0)	Dominated	N/A	−45,206 (−78,831, −8,829)	N/A	−45,206 (−78,831, −8,829)	N/A

a Costs in 2021 Canadian dollars. CI, confidence interval; ICER, incremental cost-effectiveness ratio; INMB, incremental net monetary benefit; MASC, mammary analogue secretory carcinoma; N/A, not applicable; NSCLC, non-small cell lung cancer; QALY, quality-adjusted life years.

Tumor-agnostic mean incremental costs were CAD 68,451 (95 percent confidence interval (CI): 35,466, 92,155) and USD 54,608 (95 percent CI: 28,294, 73,518), and the mean incremental QALYs were 0.13 (95 percent CI:−0.42, 0.42), resulting in a mean ICER of CAD 866,268 (95 percent CI: 180,430, 4,571,068) and USD 691,079 (95 percent CI: 143,941, 3,646,644). The INMB at a WTP of CAD 50,000/QALY was CAD −62,127 (95 percent CI: −81,677, −40,698) and USD −49,563 (95 percent CI: −65,159, −32,467) and at CAD 100,000/QALY was CAD −55,803 (95 percent CI: −89,102, −29,736) and USD −44,518 (95 percent CI: −71,083, −23,722). Across 1,000 tumor-agnostic PSA runs, 21 percent had negative QALYs and positive costs, and the probability of being cost-effective was 0.1 percent at WTP CAD 50,000/QALY and CAD 100,000/QALY.

We found substantial heterogeneity in cost-effectiveness outcomes across tumor indications. The proportion of cost-effective runs at a WTP of CAD 100,000/QALY was low, ranging from 0 (MASC) to 16 percent (NSCLC). The INMB at a WTP of both CAD 50,000/QALY and CAD 100,000/QALY was negative for all tumor indications and highest for NSCLC (CAD −23,071/USD −18,405 at CAD 100,000/QALY).

### Scenario analyses

Cost-effectiveness outcomes for the full entrectinib extrapolation scenario are presented in Supplemental Tables 6 and 7. Cost-effectiveness results are additionally provided in Figure 2 and Supplemental Figure S2, combined with the base case for comparison. Tumor-agnostic incremental costs were CAD 143,308 (95 percent CI: 73,205, 220,902) and USD 114,326 (95 percent CI: 58,400, 176,228) and incremental QALYs were 0.766 (95 percent CI: −0.141, 1.682). Across PSA runs, 5 percent of strategies had negative incremental QALYs and positive incremental costs. Compared with the base case, incremental QALYs were larger across tumor indications other than thyroid (−0.50).

Incremental outcomes for the NGS testing scenario are presented in Supplemental Tables 8–11 and Supplemental Figure S3. Testing added significant additional costs to the entrectinib arm, with tumor-agnostic incremental costs increasing to CAD 2,365,590 (95 percent CI: 1,943,339, 2,819,357) and USD 1,887,188 (95 percent CI: 1,550,330, 2,249,188). Across tumor indications, incremental costs were highest for breast (CAD 8,500,217 and USD 6,781,186), whereas they remained similar for MASC (CAD 206,149 and USD 164,459). NTRK gene fusions are highly prevalent in MASC, resulting in fewer NGS panels required to identify an NTRK-positive patient for entrectinib treatment, thereby lowering testing costs. For the full extrapolation of entrectinib effectiveness scenario, we obtained similar results. NGS costs were the key driver of greater incremental costs, regardless of the entrectinib extrapolation method.

### Decision algorithm

**Figure 3.**
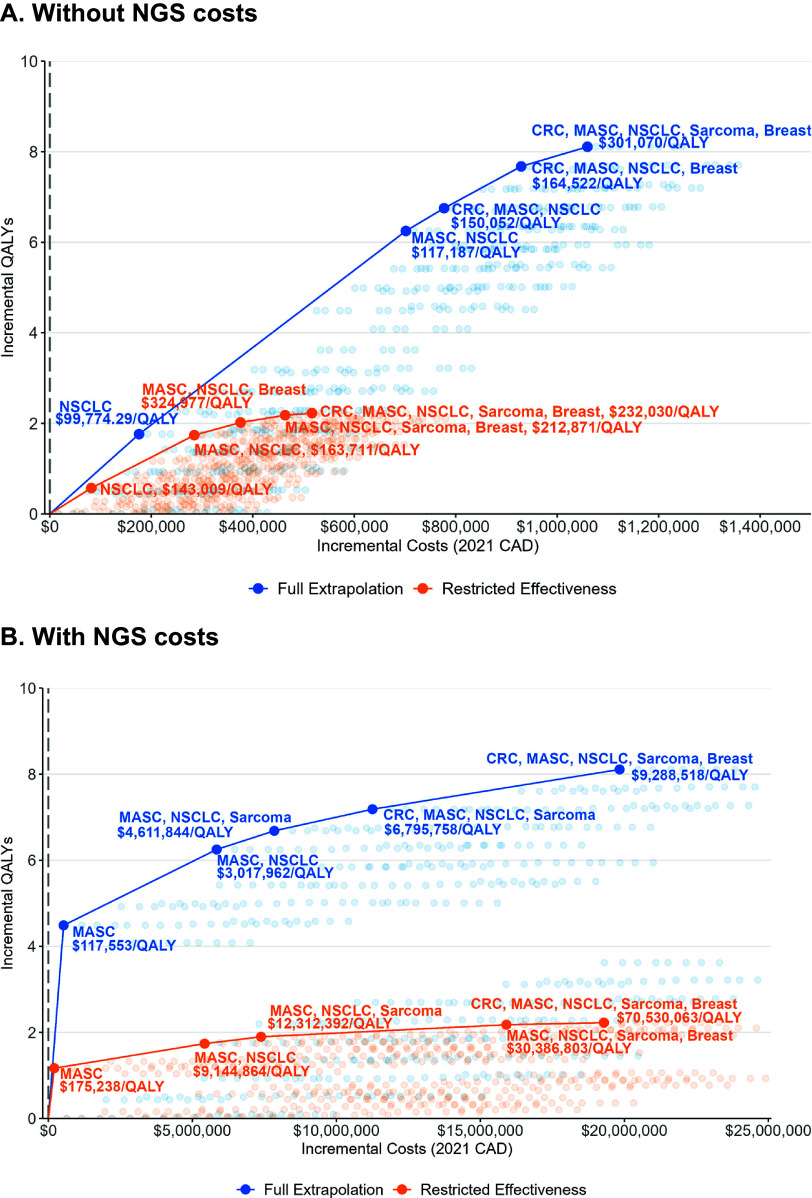
Deterministic cost-effectiveness frontier: A. without NGS costs, and B. with NGS costs. Tumor indications not included in the frontier: pancreatic, thyroid, neuroendocrine, and other. CAD, Canadian dollars; CRC, colorectal; MASC, mammary analogue secretory carcinoma; NGS, next-generation sequencing; NSCLC, non-small cell lung cancer; QALY, quality-adjusted life year.

The decision algorithm is represented by the cost-effectiveness frontier. Depending on the extrapolation assumption used, the decision algorithm recommended different strategies. Without testing costs (Figure 3A), entrectinib is the most cost-effective for reimbursement in patients with NSCLC. Under the full extrapolation scenario, entrectinib is the most cost-effective for colorectal cancer patients, and the overall QALY gains are larger. For the base case, with restricted entrectinib effectiveness, sarcoma features in the deterministic frontier as it is dominated in the probabilistic analysis but not in the deterministic analysis. With testing costs (Figure 3B), MASC represents the most cost-effective option for reimbursement for both conservative and optimistic extrapolation assumptions.

### Value of information

EVPI curves for both restricted efficacy and full extrapolation methods are included in the Supplemental File (Figures 4 and 5). For restricted efficacy, there is little benefit to reducing uncertainty across tumor indications. For the full extrapolation, there is a greater potential benefit across all tumor indications, most notably breast, colorectal, and sarcoma.

## Discussion

We developed an open-source index economic evaluation for a tumor-agnostic therapy with conditional market authorization to operationalize life-cycle HTA and support iterative decision-making. Using only publicly available information from HTA reimbursement reviews, we estimated the tumor-agnostic and tumor-specific cost-effectiveness of entrectinib compared to established therapies. Our findings aligned with CDA-AMC’s conclusions that there was no price at which entrectinib would be considered cost-effective at a WTP of CAD 50,000/QALY, with or without testing costs (16). A detailed summary of the specific results is provided in Supplemental Section 3. Our publicly available code and dashboard include all data used in this economic analysis, enabling accessible adaptation to support jurisdiction-specific reimbursement decisions. In the conduct of our analysis, we identified key operational and methodological aspects required for index economic modeling to support life-cycle HTA, with implications for uptake into regular practice by practitioners and policymakers.

Life-cycle cost-effectiveness aims to generate evidence to manage uncertainty in comparative value, prevalent in precision-oncology HTA submissions based on single-arm trials (7;8). We identified key operational gaps that limit the utility of HTA reimbursement reviews for establishing an index economic model that aids iterative decision-making. Notably, opaque public reporting, such as the lack of clearly stated tumor-specific comparators, impeded our ability to establish an index economic evaluation that would be meaningful for clinical decision-making. This uncertainty in the initial evidence base also contributed to different funding recommendations for entrectinib from Canada’s two main HTA bodies. Based on the same evidentiary package, CDA-AMC recommended reimbursement with conditions (16), whereas l’Institut national d’excellence en santé et en services sociaux, Quebec’s provincial authority on drug funding recommendations, decided against reimbursement due to ambiguity in therapeutic value (23). Discordant funding recommendations across international HTA agencies due to comparator choice have been previously noted (24). A number of governing bodies recently committed to a consistent approach to presenting redacted information (25). Previous studies also recommend increased reporting on key elements driving HTA conclusions (26;27). While national deliberations occur with access to a more detailed evidence package than what is publicly available, different conclusions emphasize the need for a clear pathway to manage change in evidentiary uncertainty for tumor-agnostic therapies (4;10;28). When there is no clinical equipoise for randomization, or where decision-makers do not reach consensus on the comparator (29), such as in the rapidly evolving landscape of precision oncology, a broader perspective for generating life-cycle decision-grade evidence remains necessary (7;8).

The need for adaptation and improved transparency to support life-cycle HTA processes is gaining recognition (30–35). In Canada and internationally, regulatory and reimbursement environments are evolving, with new initiatives such as time-limited reimbursements (36) allowing for faster and more agile decisions (32;37). Operational challenges from a lack of transparent reporting highlighted in our study concur with a European Commission-funded project producing guidance for economic evaluations in precision medicine (34;35;38). A framework for implementing life-cycle HTA also identified the need for greater transparency to achieve a consistent understanding of the initial evidence base on which regulatory and reimbursement decisions are made (10). Greater transparency in HTA reports may provide accessible information to establish a trustworthy index economic model and highlight evidence generation targets to evaluate how cost-effectiveness evolves as new evidence emerges. A recent scoping review of HTA and regulatory policies highlighted the need for better alignment on the use of emerging real-world evidence methods (39). Improving standardization and clarity of real-world evidence guidelines can inform the use of real-world data to optimize healthcare decision-making (39). A life-cycle HTA approach that limits duplicative efforts for evidence generation and adheres to recommendations on the use of real-world evidence can add meaningful value in addressing the decision problems that agile regulations seek to resolve (40–42).

Our analysis also identified methodological approaches for evaluating how uncertainty about the economic value provided by tumor-agnostic therapies evolves as new evidence emerges. By combining conservative and optimistic cost-effectiveness scenarios of long-term treatment effectiveness, we defined the limits of an envelope of uncertainty for an intervention’s potential cost-effectiveness to help guide the operationalization of life-cycle HTA. Subsequent assessments can reduce this envelope by leveraging long-term clinical outcomes data from external datasets and cross-jurisdictional real-world data sources (7;43). The bounds of the envelope provide a critical context for interpreting whether any additional benefit compared to the index evaluation demonstrates meaningful improvement compared to the standard of care, ultimately mitigating bias and leading to better decisions for patients and health systems (44). As reimbursement policies for NGS testing change over time and vary by jurisdiction and tumor indication (12;16), it is essential for a tumor-agnostic index economic evaluation to also provide the flexibility to adjust tumor-specific costs to a localized decision-making context (8). Taken together, the combined uncertainty across these key dimensions demonstrates an approach that HTA bodies can implement to establish further bounds on an envelope of uncertainty that accounts for evolving evidence of comparative effectiveness and varying policy contexts for companion diagnostic testing. In the meantime, open science analyses like ours can also help enhance consistency in frameworks and the standardization of methods to increase synergy across HTA bodies. In the long term, the rapidly evolving landscape of tumor-agnostic therapies and personalized medicine more broadly necessitates systematic changes to existing regulatory and reimbursement frameworks to address early-stage evidentiary uncertainty (28). This is particularly significant given the need to represent the heterogeneity present in an increasing number of personalized treatments.

Our decision algorithm provides a mechanism by which jurisdictions can account for heterogeneity in the potential value provided by tumor-agnostic therapies. While most international reimbursement recommendations for entrectinib, including those in Canada, are from a tumor-agnostic perspective (45), varying cost-effectiveness across tumor indications may warrant tumor-specific managed access agreements. These arrangements enable therapies to become available for a limited time while the uncertainty around demonstrated benefit is investigated (18). Prioritizing reimbursement according to the value provided can accelerate patient access to care and help ensure the long-term sustainability of health systems (7).

Our study is subject to limitations, including those previously noted by CDA-AMC in their reimbursement review of the submitted economic evaluation for entrectinib. In addition, the CDA-AMC checklist identified inadequacies in model transparency and validity in the HTA evaluation. Our code performed well in terms of accuracy across all checklist items. Methodological issues included combining first- and second-line comparator therapies, known to impact cost-effectiveness outcomes (46;47). While published HTA guidelines recommend building models that represent actual clinical care pathways, technology developers responsible for the initial HTA evidentiary package in precision oncology frequently build economic models using three-state PartSA that cannot model the complexities of current and evolving clinical care trajectories (48;49). PartSA has several known limitations (46;49) and does not typically align with HTA recommendations (1;12). Despite this, PartSA continues to inform HTA reimbursement in oncology (50), with no justification provided (48). Flexible modeling frameworks, allowing for updates to clinical care pathways and evidence of patient benefit, are needed to help the deliberation process move beyond static decision-making.

In our analysis, we chose not to use Microsoft Excel, which is currently the most commonly used software for HTA submissions (51–53), and a requirement by CDA-AMC (54). For simple analyses, Excel sheets can be managed and are easily interpretable. However, as models become more realistic, incorporating clinical pathways and real-world data, the additional complexity becomes difficult to manage (52;55). We instead used R to enable transparent, reproducible, and adaptable code, with readily available packages to aid with economic evaluations and value of information calculations (51;56). For ease of replicability and to avoid introducing spurious uncertainty, we used only the NICE reimbursement review as an additional data source and made assumptions on parameter distributions where evidence was unavailable. These assumptions may limit the utility of the probabilistic analysis and the value of information results (57). Engaging directly with HTA agencies in future research could mitigate this limitation.

### Conclusion

Index economic evaluations provide a reference point to determine how the estimated cost-effectiveness of a novel technology evolves as new evidence emerges. We developed an open-source index economic evaluation to operationalize life-cycle HTA for a conditionally authorized tumor-agnostic therapy. Our findings outline key operational and methodological considerations necessary for the development of index economic models that support life-cycle HTA, offering insights into their potential integration into regular HTA and policy decision-making processes. Open-science models and refinement of best practice guidelines for tumor-agnostic evaluations are urgently needed to address the uncertainty inherent in precision medicine and support life-cycle considerations of benefit and value, accelerating patient access to effective therapies.

## Supporting information

Cupples et al. supplementary material Cupples et al. supplementary material

## Data Availability

All data are available within this publication or in the corresponding Supplementary Files. All code and data required to reproduce the results in this manuscript are available at https://regulatory-science-lab.github.io/.

## Supplementary material

The supplementary material for this article can be found at http://doi.org/10.1017/S0266462325100111.
