# Supplementary material for: Adopting life-cycle HTA: a tumor-agnostic precision oncology index economic evaluation from publicly available reimbursement reviews
Source: Int J Technol Assess Health Care. 2025 Jun 24;41(1):e41. doi: 10.1017/S0266462325100111 (PMC12257039; doi:10.1017/S0266462325100111)
Supplement: Cupples et al. supplementary material [file S0266462325100111sup001.pdf]

Adopting life-cycle HTA: A tumor-agnostic precision oncology index economic evaluation from publicly-available reimbursement reviews

Cupples G* [1], Krebs E* [1], Weymann D [1,2], Ho C [3,4], Regier DA [1,5]

*** These authors contributed equally to this work and share first authorship.**

1. Cancer Control Research, BC Cancer Research Institute, Vancouver, BC, Canada. 2. Faculty of Health Sciences, Simon Fraser University, Vancouver, BC. 3. Department of Medical Oncology, BC Cancer, Vancouver, BC, Canada. 4. Department of Medicine, Faculty of Medicine, University of British Columbia, Vancouver, BC, Canada. 5. School of Population and Public Health, Faculty of Medicine, University of British Columbia; Vancouver, BC, Canada.

International Journal of Technology Assessment in Health Care

Contents

1. Analysis framework details and implementation 4

1.1 Partitioned survival analysis 4

1.2 Survival parameterization 4

1.3 Survival extrapolation 4

1.4 Cost inputs 4

2. Parameter uncertainty estimation for probabilistic sensitivity analysis 5

2.1 Prevalence 5

2.2 Survival 5

2.3 Utilities 5

2.4 Resource use and cost 5

3. Discussion of Analysis Results 6

Table 1: Tumor-specific Prevalence and Survival data, Including Parameter Uncertainty 7

Table 2: Tumor-specific Cost Inputs and Uncertainty Distributions 9

Table 3: Tumor-specific Number Needed to Screen to Identify Eligible Patients for Entrectinib and Associated Testing Costs [4] 10

Table 4: CDA-AMC model validation tool for the conduct of economic evaluations [5] 11

Table 5: Mean Tumor-agnostic and Tumor-specific Probabilistic Cost-effectiveness Outputs with Restricted Effectiveness of Entrectinib, in USD 14

Table 6: Mean Tumor-agnostic and Tumor-specific Probabilistic Cost-effectiveness Outputs with Full Entrectinib Extrapolation 15

Table 7: Mean Tumor-agnostic and Tumor-specific Probabilistic Cost-effectiveness Outputs with Full Entrectinib Extrapolation, in USD 16

Table 8: Mean Tumor-agnostic and Tumor-specific Probabilistic Cost-effectiveness Outputs with Restricted Effectiveness of Entrectinib, Including NGS Costs 17

Table 9: Mean Tumor-agnostic and Tumor-specific Probabilistic Cost-effectiveness Outputs with Restricted Effectiveness of Entrectinib, Including NGS Costs and in USD 18

Table 10: Mean Tumor-agnostic and Tumor-specific Probabilistic Cost-effectiveness Outputs with Full Entrectinib Extrapolation, Including NGS Costs 19

Table 11: Mean Tumor-agnostic and Tumor-specific Probabilistic Cost-effectiveness Outputs with Full Entrectinib Extrapolation, Including NGS Costs and in USD 20

Table 12: Mean Tumor-agnostic and Tumor-specific Probabilistic Cost-effectiveness Outcomes with Restricted Effectiveness of Entrectinib and Adjusted NICE Costs 21

Figure 1: Partitioned Survival Analysis Schematic. 22

Figure 2: Tumor-Specific Cost-effectiveness Plane, Without NGS Costs 23

Figure 3: Tumor-agnostic* Cost Effectiveness Plane with Restricted Effectiveness of Entrectinib, Including NGS Costs. 24
Figure 4: Tumour-agnostic* Expected Value of Perfect Information 25
Figure 5: Tumour-specific Expected Value of Perfect Information 26

1. Analysis framework details and implementation

1.1 Partitioned survival analysis

The partitioned survival analysis (PartSA) framework was developed in R, building upon the code provided by Decision Analysis in R for Technologies in Health (DARTH) [1]. The proportion of patients in one of three health states, progression-free, progressed, and death, is determined from the parameterization of survival curves. The proportion of patients in the progression-free state over time is calculated form the area under the progression-free survival curve. The proportion of patients in the progressive disease state is the area between the overall-survival and progression-free survival curves. Finally, the patients who have died is the remaining area on the plot, or one minus the proportion of patients who are both progression-free and progressed. The PartSA framework cannot distinguish between death events from progression-free or progressed disease.

1.2 Survival parameterization

Survival data was in the form of a single median survival or critical survival point, with data taken from the Canada's Drug Agency's (CDA-AMC) or the National Institute for Health and Care Excellence (NICE) health technology assessments (HTAs) for entrectinib. Exponential curves were fit though two survival points for each tumor indication, those that were provided in reimbursement reviews and via an assumption that all patients are alive and progression-free at time zero (i.e. survival proportion is one at time zero). Linear models were fit through the log transform of the data points provided and subsequently exponentiated to obtain the survival curves.

1.3 Survival extrapolation

For entrectinib, the long term efficacy was restricted to provide a conservative estimate of survival outside the trial period (Figure S1). We used within-trial efficacy during an observed period, defined as the point where 50% of the population was no longer at risk for progression or death. After the observation period, rates obtained from the comparator arm were assumed, so that the long-term treatment efficacy converges towards the comparator.

1.4 Cost inputs

Certain cost inputs were only available in 2018 British pounds from the NICE reimbursement review. These costs were converted to 2018 Canadian dollars [2], then inflated to 2021 Canadian dollars using the consumer price index for healthcare products [3]. Since the cost of goods between the United Kingdom and Canada may not be equivalent, we estimate the ratio between entrectinib list price from NICE and CDA-AMC reimbursement reviews. The NICE list price for entrectinib is £5,160 for a 30-day treatment cycle, and the CDA's list price is CAD 8007.72 for a 28 day treatment cycle, leading to a cost ratio of 0.913. Since only a 9% reduction in NICE costs would be required, these changes would not impact the cost-effectiveness decision. Base case results, including this cost adjustment, are provided in **Table 12**.

Comparator therapy costs were available for a number of standard care treatments per tumor indication in the CDA-AMC reimbursement review. We were unable to verify common treatments between the drug costs and survival estimates from the NICE reimbursement review. To obtain treatment costs per tumor indication, we took an average of the reported therapy costs. An average of multiple therapy admin costs was reported in the NICE reimbursement review, and used in our analysis. Drug administration costs were included for infusion-based therapies only, no costs were attributed for oral therapies. Next-generation sequencing unit costs, estimated from the number needed to screen to identify a single NTRK+ patient, were $1,400 [4].

2. Parameter uncertainty estimation for probabilistic sensitivity analysis

The CDA-AMC and NICE reimbursement reviews for entrectinib were used to characterize parameter uncertainty where information was available. In general, the reports provide only basic information which created challenges in determining both the distribution type and parameters needed to characterize each distribution.

2.1 Prevalence

No information was available to characterize the uncertainty in prevalence data in the CDA-AMC reimbursement review. We used a Dirichlet distribution, so that the prevalence of each tumor indication was dependent on the overall cancer prevalence used to calculate the weighted average. Using separate information about overall and neurotrophic tyrosine receptor kinase (NTRK) gene fusion prevalence would provide an opportunity to investigate scenarios where tumor indications are not always represented in the analysis.

2.2 Survival

Uncertainty for entrectinib survival estimates was provided in CDA-AMC's reimbursement review. The uncertainty was characterized based on the type of estimate available. For tumor indications with median survival data, we used a truncated normal distribution. In these cases, the standard deviation (SD) was calculated from the standard error and trial population. When the median was not achieved during the trial, and a critical point was instead used, a beta distribution is assumed around the survival proportion at the identified time point.

The survival estimates for the comparator arm, provided in the NICE reimbursement review, did not contain information to characterize uncertainty. We assumed a truncated normal distribution throughout, as all survival data were presented as medians. The distribution was truncated at zero, to avoid negative survival estimates. Since no other information was available to characterize uncertainty, the standard deviation was assumed equal to the median. Survival data is provided in detail in Table S1, where truncated normal distributions are in the form (mean, SD), and Beta distributions are in the form (α, β).

2.3 Utilities

Without additional information, utilities were assumed to vary according to a uniform distribution of ±10%. This value was chosen to ensure that for all model runs included in the probabilistic analysis, quality of life for progressive disease remained lower than progression-free disease.

2.4 Resource use and cost

A uniform distribution was also assumed for all varying costs. A number of costs were assumed to be fixed in the analysis, including drug costs (both entrectinib and the comparator), and drug administration costs for entrectinib. All other costs were assumed to vary by ±20%. Tumor-specific costs are detailed in Table S2 and S3, all other costs are included in the main text, Table 1.

3. Discussion of Analysis Results

We developed an index economic evaluation to operationalize life-cycle economic evaluations and establish how estimated cost effectiveness evolves as new evidence emerges. As a tumor-agnostic therapy, entrectinib had a 0.1 percent probability of being cost effective at a WTP of both CAD 50,000/QALY and CAD 100,000/QALY, along with a 20 percent probability of being dominated by standard care. In a tumor-specific setting, we found substantial heterogeneity across tumor indications, with treatment for NSCLC providing the greatest value (INMB CAD -23,071 at CAD 100,000/QALY; 95% CI: CAD -74,028, 36,807) (USD -18,405, 95%CI: -59,057, 29,363).

For the scenario with full extrapolation of entrectinib effectiveness, cost-effectiveness results were also heterogeneous across tumor indications. Compared with the base case, incremental QALYs were larger for the pooled analysis, across all tumour indications other than Thyroid (-0.36 vs. -0.50). However we see a broader spread of incremental costs and QALYs, likely due to the propagation of uncertainty through the extrapolation of entrectinib effectiveness. The need to reimburse companion diagnostic tests greatly impacted the potential value of entrectinib for all tumor indications other than MASC (INMB CAD -93,674 at CAD 100,000/QALY) (USD -73,416), where NTRK fusion prevalence is highest. Overall, our findings aligned with CDA-AMC's conclusions that there was no price at which entrectinib would be considered cost effective at a WTP of CAD 50,000/QALY, with or without testing costs [4]. However, substantial uncertainty in our point estimates remains.

A European Commission-funded project producing guidance for economic evaluations in precision medicine recently published a pilot economic evaluation of entrectinib [5-7]. The model closely followed these guidelines, drawing on population-level real-world data from the Netherlands to parameterize survival data for NTRK-negative patients [8]. The intervention arm comprised NTRK testing for all patients with locally advanced or metastatic solid tumors, and subsequent treatment with entrectinib or standard care based on NTRK status. Since NTRK gene fusions are rare, the proportion of entrectinib-treated patients in the intervention arm was very small, masking incremental effects on costs and benefits. Additionally, the authors utilized pooled survival data for entrectinib, which is not reflective of the heterogeneity in effectiveness. We incorporated testing costs via prevalence-based estimates of the number needed to screen to identify one NTRK+ patient, and estimated tumor-specific cost effectiveness from stratified entrectinib data.

Table 1: Tumor-specific Prevalence and Survival data, Including Parameter Uncertainty

Parameter	Value	Distribution	Distribution parameters	Source
Breast				
Prevalence (%)	0.40	Dirichlet[a]	N/A	CDA-AMC
OS (months)	23.90	Truncated normal	(23.90, 29.73)	CDA-AMC
OS proportion	0.50	Fixed	-	CDA-AMC
PFS (months)	10.10	Truncated normal	(10.10, 14.86)	CDA-AMC
PFS proportion	0.50	Fixed	-	CDA-AMC
End of observation (months)	10	Fixed	-	CDA-AMC
Comparator OS (months)	12.18	Truncated normal	(12.18, 12.18)	NICE
Comparator PFS (months)	3.03	Truncated normal	(3.03, 3.03)	NICE
Colorectal				
Prevalence (%)	8.30	Dirichlet[a]	N/A	CDA-AMC
OS (months)	16	Truncated normal	(16, 33.31)	CDA-AMC
OS proportion	0.50	Fixed		CDA-AMC
PFS (months)	2.40	Truncated normal	(2.40, 33.31)	CDA-AMC
PFS proportion	0.50	Fixed	-	CDA-AMC
End of observation (months)	2.50	Fixed	-	CDA-AMC
Comparator OS (months)	9.07	Truncated normal	(9.07, 9.07)	NICE
Comparator PFS (months)	2.63	Truncated normal	(2.63, 2.63)	NICE
MASC				
Prevalence (%)	9.50	Dirichlet[a]	N/A	CDA-AMC
OS (months)	27	Fixed	-	CDA-AMC
OS proportion	0.92	Beta	(11.96, 1.04)	CDA-AMC
PFS (months)	21	Fixed	-	CDA-AMC
PFS proportion	0.70	Beta	(9.10, 3.90)	CDA-AMC
End of observation (months)	21	Fixed	-	CDA-AMC
Comparator OS (months)	13.80	Truncated normal	(13.80, 13.80)	NICE
Comparator PFS (months)	4.35	Truncated normal	(4.35, 4.35)	NICE
NSCLC				
Prevalence (%)	20.20	Dirichlet[a]	N/A	CDA-AMC
OS (months)	15	Fixed	-	CDA-AMC
OS proportion	0.75	Beta	(9.75, 3.25)	CDA-AMC
PFS (months)	14	Truncated normal	(14, 10.20)	CDA-AMC
PFS proportion	0.50	Fixed	-	CDA-AMC
End of observation (months)	15	Fixed	-	CDA-AMC
Comparator OS (months)	10.65	Truncated normal	(10.65, 10.65)	NICE
Comparator PFS (months)	3.75	Truncated normal	(3.75, 3.75)	NICE
Neuroendocrine				
Prevalence (%)	0.25	Dirichlet[a]	N/A	CDA-AMC
OS (months)	39.61	Truncated normal	(39.61, 39.61)	NICE
OS proportion	0.50	Fixed	-	NICE
PFS (months)	8.03	Truncated normal	(8.03, 8.03)	NICE

PFS proportion	0.50	Fixed	-	NICE
End of observation (months)	N/A	Fixed	-	N/A
Comparator OS (months)	39.61	Truncated normal	(39.61, 39.61)	NICE
Comparator PFS (months)	8.03	Truncated normal	(8.03, 8.03)	NICE
Pancreatic				
Prevalence (%)	10	Dirichlet[a]	N/A	CDA-AMC
OS (months)	8.80	Truncated normal	(8.80, 8.80)	NICE
OS proportion	0.50	Fixed	-	NICE
PFS (months)	5.20	Truncated normal	(5.20, 5.20)	NICE
PFS proportion	0.50	Fixed	-	NICE
End of observation (months)	N/A	N/A	N/A	N/A
Comparator OS (months)	8.80	Truncated normal	(8.80, 8.80)	NICE
Comparator PFS (months)	5.20	Truncated normal	(5.20, 5.20)	NICE
Sarcoma				
Prevalence (%)	0.60	Dirichlet[a]	N/A	CDA-AMC
OS (months)	19	Truncated normal	(19, 8.20)	CDA-AMC
OS proportion	0.50	Fixed	-	CDA-AMC
PFS (months)	10	Truncated normal	(10, 4.76)	CDA-AMC
PFS proportion	0.50	Fixed	-	CDA-AMC
End of observation (months)	10	Fixed	-	CDA-AMC
Comparator OS (months)	14.30	Truncated normal	(14.30, 14.30)	NICE
Comparator PFS (months)	3.90	Truncated normal	(3.90, 3.90)	NICE
Thyroid				
Prevalence (%)	23.80	Dirichlet[a]	N/A	CDA-AMC
OS (months)	22	Truncated normal	(22, 19.80)	CDA-AMC
OS proportion	0.50	Fixed	-	CDA-AMC
PFS (months)	11.80	Truncated normal	(11.80, 8.10)	CDA-AMC
PFS proportion	0.50	Fixed	-	CDA-AMC
End of observation (months)	12	Fixed	-	CDA-AMC
Comparator OS (months)	30.95	Truncated normal	(30.95, 30.95)	NICE
Comparator PFS (months)	4.55	Truncated normal	(4.55, 4.55)	NICE
Other				
Prevalence (%)	27.10	Dirichlet[a]	N/A	CDA-AMC
OS (months)	17.23	Truncated normal	(17.23, 17.23)	NICE
OS proportion	0.50	Fixed		NICE
PFS (months)	4.35	Truncated normal	(4.35, 4.35)	NICE
PFS proportion	0.50	Fixed	-	NICE
End of observation (months)	N/A	N/A	N/A	N/A
Comparator OS (months)	17.23	Truncated normal	(17.23, 17.23)	NICE
Comparator PFS (months)	4.35	Truncated normal	(4.35, 4.35)	NICE

[a] Distribution assumed. CAD: Canadian dollars; CDA-AMC: Canada's Drug Agency; MASC: mammary analogue secretory carcinoma; N/A: not applicable; NICE: National Institute for Health and Care Excellence; NGS: next-generation sequencing; NSCLC: non-small cell lung cancer; OS: overall survival; PFS: progression-free survival; PSA: probabilistic sensitivity analysis.

Table 2: Tumor-specific Cost Inputs and Uncertainty Distributions

	Comparator Drug			Comparator Administration			Non-Cancer Care		
Tumor Indication	Value	PSA	Source	Value	PSA[a]	Source	Value	PSA[a]	Source
Breast	552.32 (USD 441.42)	fixed	CDA-AMC	139.01 (USD 110.90)	±20%	NICE	264.36 (USD 210.90)	±20%	CDA-AMC
Colorectal	1,172.78 (USD 935.60)	fixed	CDA-AMC	116.94 (USD 93.29)	±20%	NICE	266.29 (USD 212.44)	±20%	CDA-AMC
MASC	761.30 (USD 607.34)	fixed	CDA-AMC	0	±20%	NICE	242.92 (USD 193.79)	±20%	CDA-AMC
NSCLC	2,386.45 (USD 1903.83)	fixed	CDA-AMC	142.52 (USD 113.70)	±20%	NICE	276.93 (USD 220.93)	±20%	CDA-AMC
Neuroendocrine	47.02 (USD 37.51)	fixed	CDA-AMC	3.07 (USD 2.45)	±20%	NICE	242.78 (USD 193.68)	±20%	CDA-AMC
Pancreatic	1,729.74 (USD 1,379.93)	fixed	CDA-AMC	183.30 (USD 146.23)	±20%	NICE	315.38 (USD 251.60)	±20%	CDA-AMC
Sarcoma	443.26 (USD 353.62)	fixed	CDA-AMC	139.46 (USD 111.26)	±20%	NICE	243.02 (USD 193.87)	±20%	CDA-AMC
Thyroid	1,070.61 (USD 854.10)	fixed	CDA-AMC	0	±20%	NICE	142.88 (USD 113.98)	±20%	CDA-AMC
Other[b]	1,484.58 (USD 1,184.35)	fixed	CDA-AMC	90.54 (USD 72.23)	±20%	NICE	232.42 (USD 185.42)	±20%	CDA-AMC

[a] Uniform distribution assumed. [b] Values are calculated as weighted average of other tumor indications. CAD: Canadian dollars; CDA-AMC: Canada's Drug Agency; NICE: National Institute for Health and Care Excellence; PSA: probabilistic sensitivity analysis.

Table 3: Tumor-specific Number Needed to Screen to Identify Eligible Patients for Entrectinib and Associated Testing Costs [4]

Tumor indication	Number needed to screen with NGS to find one NTRK fusion (95% CI)	Testing costs, 2021 Canadian dollars	PSA[a]
Breast – secretory	1.1 (1, 2)	8,429,497[b] (USD 6,724,768)	±20%
Breast – non-secretory	6,036 (207, 32,548)		±20%
Colorectal	2,382 (91, 12,267)	3,335,467 (USD 2,660,923)	±20%
MASC	1.2 (1, 2)	1,643 (1,311)	±20%
NSCLC	3,671 (124, 18,269)	5,139,639 (USD 4,100,231)	±20%
Neuroendocrine	739 (374, 1,232)	1,034,081 (USD 824,955)	±20%
Pancreatic	1,564 (71, 6,959)	2,189,416 (USD 1,746,642)	±20%
Sarcoma	1,336 (138, 6,741)	1,870,088 (USD 1,491,893)	±20%
Thyroid	1,698 (11, 4,310)	2,377,651 (USD 1,896,810)	±20%
Cancer of unknown primary	385	585,801[b] (USD 467,322)	±20%
Cholangiocarcinoma	500		
Endometrial	526		
Head and Neck	526		
Neuroblastoma	192		
Ovarian	323		

[a] Distribution assumed. [b] Weighted average of testing costs assumed. MASC: mammary analogue secretory carcinoma; NGS: next-generation sequencing; NSCLC: non-small cell lung cancer; NTRK: neurotrophic tyrosine receptor kinase; PSA: probabilistic sensitivity analysis.

Table 4: CDA-AMC model validation tool for the conduct of economic evaluations [9]

Item	Description	Yes	No	NA
1	The model built is reflective of the stated population that the decision problem applies to.		x	
2	The model can examine key subgroups within the population of interest.	x		
3	The model assesses all comparators used to currently treat the stated population.		x	
4	The model incorporates costs that are consistent with the specified perspective of the analysis.	x		
5	The model assesses all outcomes deemed important by clinicians and patients.		x	
6	The structure of the model (i.e., the process and clinical pathway) has been validated by clinical experts.		x	
7	The model follows previous models in this clinical area or justification has been provided about why the model structure differs from previous models.<br>[Select NA if there are no previous models in this clinical area.]		x	
8	Time spent in each health state, for each technology assessed, can be extracted from the model.<br>[Select NA if the model does not utilize health states (e.g., a decision tree) and skip item 9.]	x		
9	The model output matches the evidence provided to support time spent in health states among technologies.		x	
10	If clinical events are modelled (i.e., hospitalizations, exacerbations, strokes, hip fractures), the number of events for each technology can be extracted from the model.<br>[Select NA if clinical events are not relevant to the decision problem and skip item 11.]			x
11	Model output matches the evidence provided to support the number of clinical events across technologies.			x
12	The impact of adverse events on health outcomes and costs can be extracted from the model.<br>[Select NA if adverse events are not relevant to the decision problem and skip item 13.]		x	
13	The model output matches evidence provided for adverse event type and frequency from the evidence.			x
14	Life-years are reported as a result within the model.	X		
15	The impact the evaluated technologies has on mortality is clear.<br>[Select NA if there are no differences in mortality and skip items 16, 17, and 18.]	X		
16	If differences in mortality are noted in Item 15, select the reasons for differing mortality in the model (more than 1 reason can be selected):<br>a. Duration of time spent in health states lead to higher mortality risk.<br>b. There is a difference in the frequency of fatal clinical events.<br>c. There is a difference in the frequency of fatal adverse events.<br>d. Direct impact on risk of death, not stated previously, has been modelled (i.e., direct modelling of overall survival from the trial).	d		
17	If there are mortality differences between technologies, the model can extract which of the reasons from item 16 has the largest impact on incremental life-years.		x	
18	Model output matches evidence regarding mortality rates between different technologies.		x	
19	It is clear that the model does not utilize technology-specific utilities.	x		
20	Based on the results from the submitted model, it can be determined which of the following has the largest impact on cost-effectiveness conclusions: time spent in health states, number of clinical events occurring, adverse events, and mortality.		x	
21	The model distinguishes data that are based on extrapolation methods (i.e., using parametric survival analysis).<br>[Select NA if no extrapolation is required.]	x		
22	The model time horizon can be adjusted for just the period for which there are clinical data available.<br>[Select NA if the data cover the full period for which clinical data are available.]	x		
23	If the model incorporates both direct and indirect effects, it is clear how double counting has been avoided (e.g., a direct effect applies to mortality through applying a hazard ratio to overall survival and an indirect effect is applied to the probability of an event or transition that is associated with a mortality risk).<br>[Select NA if only direct or indirect effects are included.]			x

24	Does the modelled relationship between surrogate outcomes and final outcomes (quality of life and mortality) match the evidence presented?<br>[Select NA if no surrogate outcomes are used.]			**x**
25	The model allows flexibility to explore waning of treatment effects OR evidence and rationale is provided that suggests treatment effects are permanent and enduring.<br>[Select NA if no extrapolation of treatment effect is required.]	**x**		
26	The model can access the deterministic result and the results from single Monte Carlo simulations.	**x**		
27	A clear trace can be identified that links all input parameters to final outcomes (i.e., only input parameters are hard coded).	**x**		
28	Macros are exclusively related to first- or second-order simulation and model navigation (exclusive to models built in Microsoft Excel).			**x**
29	You can set the effectiveness of different technologies such that QALY estimates are equal.	**x**		
30	When you set effectiveness values to be extremely in favour of or against 1 technology, this leads to substantially greater or reduced QALY estimates.	**x**		
31	When you set effectiveness values for 1 technology to be slightly improved or reduced, this leads to greater or reduced QALY estimates.	**x**		
32	When you increase mortality risk for each health state or event, this leads to lower QALYs and life-years for all technologies.	**x**		
33	When you reduce mortality risk for each health state or event, this leads to greater QALYs and life-years for all technologies.	**x**		
34	When you increase baseline risks of events, this leads to lower QALYs for all technologies.	**x**		
35	When you reduce baseline risks of events, this leads to higher QALYs for all technologies.	**x**		
36	When you set mortality to be zero (i.e., patients do not enter the death state), life-years are identical across technologies.	**x**		
37	When you increase the cost of a technology, the only output impacted is the total lifetime costs for strategies that include that technology; likewise, there is no effect on QALYs or life-years.	**x**		
38	When you set all utilities to 1 and all disutilities to zero, the estimated QALYs are equivalent to life-years.	**x**		
39	For evaluations with a time horizon greater than 1 year, when you set the discount rate to 0%, the costs and QALYs for all interventions increase.<br>[Select NA if the time horizon is shorter than 1 year because discounting is only relevant for models with time horizons longer than 1 year.]	**x**		
40	For evaluations with a time horizon greater than 1 year, if you increase the discount rate, the costs and QALYs for all interventions decrease.<br>[Select NA if the time horizon is shorter than 1 year because discounting is only relevant for models with time horizons longer than 1 year.]	**x**		
41	When you reduce the time horizon of the evaluation (the period costs and QALYs are estimated) this leads to lower estimated costs and QALYs for all interventions.	**x**		
42	It is possible to switch the inputs for 2 technologies and get the same results as before, meaning by changing the inputs (effectiveness, costs, QALYs), the model structure for any decision alternative can be used to model any other decision alternative.			**x**
43	You can calculate the correlation between the costs and QALYs for different technologies across the Monte Carlo simulation replications.			**x**
44	Based on the results of the Monte Carlo simulation, there is a strong correlation between the estimates of costs (i.e., the estimated costs from each replication) for different technologies.	**x**		
45	Based on the results of the Monte Carlo simulation, there is a strong correlation between the estimates of QALYs (i.e., the estimated QALYs from each replication) for different technologies.	**x**		
46	The results of the deterministic analysis are broadly in line with the results of the probabilistic analysis. Justification is provided about why deterministic and probabilistic results are different.	**x**		
47	You can work backward from the results of the model to the location where inputs are entered.	**x**		

	[For example, if you take the total costs associated with an intervention, can you work back from this value to determine how it was estimated and what inputs were used to derive this value?]			
48	You can work forward from the location where inputs are entered to the results of a single Monte Carlo simulation.<br>[For example, if you take a random input into the model (e.g., technology cost), can you trace how this input influences costs and or QALYs in the model?]	**x**		
49	The use of the following functions limit model transparency, are inefficient, and are not required:<br>• IFERROR, IFNA, ISERROR, ISERR, or ISNA<br>• CHOOSE, INDIRECT, OFFSET, and INDEX<br>The model makes no or limited use of these statements.			**x**
50	The model has no hidden sheets, rows, and columns.			**x**
51	The model is free of user-created formulas embedded within VBA macros.			**x**
52	Parameters are not reset to default values after macros (e.g., for a Monte Carlo simulation) are run.	**x**		
53	All input parameters that influence model results are provided in a transparent manner, preferably in a single worksheet.	**x**		

Table 5: Mean Tumor-agnostic and Tumor-specific Probabilistic Cost-effectiveness Outputs with Restricted Effectiveness of Entrectinib, in USD

	Incremental cost, USD Mean (95% CI)	Incremental QALYs Mean (95% CI)	ICER, USD/QALY Mean (95% CI)	Mean INMB at CAD 50,000/QALY, USD	Mean INMB at CAD 100,000/QALY, USD
Tumor-agnostic	55,623 (30,109, 75,090)	0.126 (-0.419, 0.421)	691,079 (143,941, 3,646,644)	-50,349 (-65,229, -32,582)	-45,075 (-72,958, -27,182)
MASC	162,831 (131,714, 200,446)	1.125 (0.634, 1.432)	152,664 (109,686, 254,886)	-118,027 (-157,176, -89,455)	-73,223 (-118,514, -40,370)
NSCLC	60,325 (-6,181, 100,117)	0.494 (-0.134, 0.873)	152,340 (2,095, 567,095)	-40,044 (-75,457, 13,294)	-19,762 (-63,004, 30,551)
Breast	66,827 (-440, 101,059)	0.173 (-1.12, 0.578)	1,026,162 (18,344, 2,003,600)	-61,240 (-87,356, -26,312)	-55,651 (-94,601, -30,354)
Colorectal	40,425 (3,588, 61,933)	0.037 (-0.437, 0.224)	1,177,885 (47,196, 4,156,028)	-38,895 (-60,782, -14,519)	-37,130 (-61,462, -10,694)
Sarcoma	62,974 (13,362, 114,122)	-0.008 (-1.064, 0.488)	Dominated	-63,300 (-89,157, -40,070)	-63,627 (-110,279, -31,564)
Thyroid	49,415 (-45,035, 88,132)	-0.357 (-2.592, 0.591)	Dominated	-63,485 (-95,550, -23,764)	-77,556 (-170,164, -25,923)
Pancreatic	19,294 (1,671, 51,939)	0 (0, 0)	Dominated	-19,294 (-51,939, -1,671)	-19,294 (-51,939, -1,671)
Neuro-endocrine	111,868 (31,114, 216,282)	0 (0, 0)	Dominated	-111,868 (-216,282, -31,114)	-111,868 (-216,282, -31,114)
Other	36,427 (6,809, 61,122)	0 (0, 0)	Dominated	-36,247 (-61,122, -6,809)	-36,247 (-61,122, -6,809)

CI: Confidence interval; ICER: incremental cost-effectiveness ratio; INMB: incremental net monetary benefit; MASC: mammary analogue secretory carcinoma; NA: not applicable; NSCLC: non-small cell lung cancer; QALY: Quality adjusted life years; USD: United States Dollar (2021).

Table 6: Mean Tumor-agnostic and Tumor-specific Probabilistic Cost-effectiveness Outputs with Full Entrectinib Extrapolation

	Incremental cost, CAD Mean (95% CI)	Incremental QALYs Mean (95% CI)	ICER, CAD/QALY Mean (95% CI)	% Dominated	INMB at CAD 50,000/QALY, CAD Mean (95% CI)	INMB at CAD 100,000/QALY, CAD Mean (95% CI)	% CE at CAD 50,000/ 100,000
Tumor-agnostic	143,308 (73,205, 220,902)	0.766 (-0.141, 1.682)	306,645 (99,437, 1,051,022)	5	-104,986 (-161,810, -55,640)	-66,664 (-133,079, -68)	0/2.5
MASC	546,617 (310,177, 842,722)	4.36 (2.165, 6.20)	131,349 (74,963, 222,341)	0	-328,396 (-505,960, -117,090)	-110,174 (-363,953, 128,606)	0/21.6
NSCLC	184,981 (4,089, 382,643)	1.77 (-0.165, 4.115)	139,451 (24,718, 453,190)	3.3	-96,469 (-262,747, 35,663)	-7,957 (-176,368, 175,846)	9.6/44.9
Colorectal	189,302 (-31,757, 498,926)	1.299 (-1.089, 3.568)	188,329 (23,668, 641,972)	11.8	-124,329 (-359,042, -4,431)	-59,357 (-214,812, 110,273)	5.6/25.9
Breast	178,869 (-14,534, 401,096)	1.122 (-1.464, 3.324)	242,087 (13,125, 818,088)	14.9	-122,767 (-288,479, -24,008)	-66,665 (-214,272, 84,522)	4.8/25.1
Sarcoma	111221 (-4,419, 217,353)	0.149 (-1.687, 1.773)	615,180 (13,868, 3,074,884)	39	-103,768 (-178,177, -45,815)	-96,315 (-207,404, 452)	3/5.7
Thyroid	93,661 (-64,115, 281,060)	-0.498 (-3.11, 2.232)	Dominated	50.7	-118,545 (-251,549, -17,153)	-143,430 (-311,993, 42,871)	14.3/20.6
Pancreatic	23,154 (1,662, 63,438)	0 (0, 0)	Dominated	N/A	-23,154 (-63,438, -1,662)	-23,154 (-63,438, -1,662)	N/A
Neuroendocrine	138,896 (41,784, 273,467)	0 (0, 0)	Dominated	N/A	-138,896 (-273,467, -41,784)	-138,896 (-273,467, -41,784)	N/A
Other	45,206 (8,829, 78,831)	0 (0, 0)	Dominated	N/A	-45,206 (-78,831, -8,829)	-45,206 (-78,831, -8,829)	N/A

CAD: 2021 Canadian dollars; CE; cost effective; CI: confidence interval; ICER: incremental cost-effectives ratio; MASC: mammary analogue secretory carcinoma; N/A: not applicable; NSCLC: non-small cell lung cancer; QALY: quality-adjusted life year.

Table 7: Mean Tumor-agnostic and Tumor-specific Probabilistic Cost-effectiveness Outputs with Full Entrectinib Extrapolation, in USD

	Incremental cost, USD Mean (95% CI)	Incremental QALYs Mean (95% CI)	ICER, USD/QALY Mean (95% CI)	INMB at CAD 50,000/QALY, USD Mean (95% CI)	INMB at CAD 100,000/QALY, USD Mean (95% CI)
Tumor-agnostic	114,326 (58,400, 176,228)	0.766 (-0.141, 1.682)	244,631 (79,327, 838,470)	-83,754 (-129,087, -44,388)	-53,182 (-106,166, -54)
MASC	436,073 (247,449, 672,295)	4.36 (2.165, 6.20)	104,786 (59,803, 177,376)	-261,983 (-403,638, -93,410)	-87,893 (-290,349, 102,598)
NSCLC	147,572 (3,262, 305,260)	1.77 (-0.165, 4.115)	111,249 (19,719, 361,540)	-76,960 (-209,611, 28,451)	-6,348 (-140,700, 140,284)
Colorectal	151,019 (-25,335, 398,026)	1.299 (-1.089, 3.568)	150,243 (18,882, 512,144)	-99,185 (-286,432, -3,535)	-47,353 (-171,370, 87,972)
Breast	142,696 (-11,595, 319,981)	1.122 (-1.464, 3.324)	193,129 (10,471, 652,643)	-97,939 (-230,139, -19,153)	-53,183 (-170,939, 67,429)
Sarcoma	88,728 (-3,525, 173,397)	0.149 (-1.687, 1.773)	490,770 (11,063, 2,453,039)	-82,783 (-142,144, -36,550)	-76,837 (-165,460, 361)
Thyroid	74,720 (-51,149, 224,220)	-0.498 (-3.11, 2.232)	Dominated	-94,571 (-200,677, -13,684)	-114,424 (-248,897, 34,201)
Pancreatic	18,471 (1,326, 50,609)	0 (0, 0)	Dominated	-18,471 (-50,609, -1,326)	-18,471 (-50,609, -1,326)
Neuroendocrine	110,807 (33,334, 218,163)	0 (0, 0)	Dominated	-110,807 (-218,163, -33,334)	-110,807 (-218,163, -33,334)
Other	36,064 (7,043, 62,889)	0 (0, 0)	Dominated	-36,064 (-62,889, -7,043)	-36,064 (-62,889, -7,043)

CI: confidence interval; ICER: incremental cost-effectives ratio; INMB: incremental net monetary benefit; MASC: mammary analogue secretory carcinoma; N/A: not applicable; NSCLC: non-small cell lung cancer; QALY: quality-adjusted life year; USD: United States Dollar (2021).

Table 8: Mean Tumor-agnostic and Tumor-specific Probabilistic Cost-effectiveness Outputs with Restricted Effectiveness of Entrectinib, Including NGS Costs

	Incremental cost, CAD Mean (95% CI)	Incremental QALYs Mean (95% CI)	ICER, CAD/QALY Mean (95% CI)	% Dominated	INMB at CAD 50,000/QALY, CAD	INMB at CAD 100,000/QALY, CAD	% CE at CAD 50,000/ 100,000
Tumor-agnostic	2,365,590 (1,943,339, 2,819,357)	0.126 (-0.419, 0.421)	32,482,265 (5,471,235, 155,452,031)	20.6	-2,359,266 (-2,806,707, -1,936,582)	-2,352,942 (-2,799,072, -1,929,250)	0/0
MASC	206,149 (171,371, 252,854)	1.125 (0.634, 1.432)	192,916 (138,535, 322,297)	0	-149,911 (-196,712, -114,874)	-93,674 (-150,975, -51,457)	0/0
NSCLC	5,193,127 (4,223,266, 6,174,506)	0.494 (-0.134, 0.873)	17,849,903 (5,558,907, 90,496,188)	4.9	-5,168,425 (-6,146,204, -4,201,689)	-5,143,723 (-6,124,662, -4,174,869)	0/0
Breast	8,500,217 (6,904,600, 10,120,546)	0.173 (-1.12, 0.578)	128,327,944 (13,352,315, 319,229,359)	19.7	-8,491,587 (-10,104,430, -6,890,411)	-8,482,957 (-10,098,079, -6,878,621)	0/0
Colorectal	3,375,016 (2,757,638, 4,013,031)	0.037 (-0.437, 0.224)	92,018,892 (13,676,152, 304,216,706)	25.5	-3,373,149 (-4,008,360, -2,758,972)	-3,371,283 (-4,005,527, -2,754,084)	0/0
Sarcoma	1,954,621 (1,584,302, 2,305,924)	-0.008 (-1.064, 0.488)	Dominated	40.1	-1,955,015 (-2,301,216, -1,591,753)	-1,955,409 (-2,302,595, -1,588,340)	0/0
Thyroid	2,443,419 (1,985,652, 2,894,763)	-0.357 (-2.592, 0.591)	Dominated	63.6	-2,461,255 (-2,904,475, -2,007,748)	-2,479,091 (-2,931,788, -2,006,702)	0/0
Pancreatic	2,212,179 (1,792,905, 2,629,562)	0 (0, 0)	Dominated	N/A	-2,212,179 (-2,629,562, -1,792,905)	-2,212,179 (-2,629,562, -1,792,905)	0/0
Neuroendocrine	1,168,030 (942,444, 1,404,418)	0 (0, 0)	Dominated	N/A	-1,168,030 (-1,404,418, -942,444)	-1,168,030 (-1,404,418, -942,444)	0/0
Other	628,949 (513,701, 747,997)	0 (0, 0)	Dominated	N/A	-628,949 (-747,997, -513,701)	-628,949 (-747,997, -513,701)	0/0

CAD: 2021 Canadian dollars; CE; cost effective; CI: confidence interval; ICER: incremental cost-effectives ratio; MASC: mammary analogue secretory carcinoma; N/A: not applicable; NSCLC: non-small cell lung cancer; QALY: quality-adjusted life year.

Table 9: Mean Tumor-agnostic and Tumor-specific Probabilistic Cost-effectiveness Outputs with Restricted Effectiveness of Entrectinib, Including NGS Costs and in USD

	Incremental cost, USD Mean (95% CI)	Incremental QALYs Mean (95% CI)	ICER, USD/QALY Mean (95% CI)	INMB at CAD 50,000/QALY, USD	INMB at CAD 100,000/QALY, USD
Tumor-agnostic	1,887,188 (1,550,330, 2,249,188)	0.126 (-0.419, 0.421)	25,913,255 (4,364,767, 124,014,385)	-1,882,143 (-2,239,096, -1,544,940)	-1,877,098 (-2,233,005, -1,539,091)
MASC	164,459 (136,714, 201,718)	1.125 (0.634, 1.432)	153,902 (110,519, 257,118)	-119,594 (-156,930, -91,643)	-74,730 (-120,443, -41,051)
NSCLC	4,142,901 (3,369,179, 4,925,813)	0.494 (-0.134, 0.873)	14,240,050 (4,434,708, 72,194,805)	-4,123,195 (-4,903,234, -3,351,966)	-4,103,489 (-4,886,049, -3,330,570)
Breast	6,781,186 (5,508,257, 8,073,830)	0.173 (-1.12, 0.578)	102,375,703 (10,652,026, 254,670,410)	-6,774,302 (-8,060,973, -5,496,937)	-6,767,417 (-8,055,907, -5,487,532)
Colorectal	2,692,474 (2,199,951, 3,201,461)	0.037 (-0.437, 0.224)	73,409,567 (10,910,373, 242,693,822)	-2,690,984 (-3,197,734, -2,201,015)	-2,689,496 (-3,195,474, -2,197,115)
Sarcoma	1,559,331 (1,263,903, 1,839,588)	-0.008 (-1.064, 0.488)	Dominated	-1,559,645 (-1,835,832, -1,269,847)	-1,559,959 (-1,836,933, -1,267,124)
Thyroid	1,949,277 (1,584,086, 2,309,344)	-0.357 (-2.592, 0.591)	Dominated	-1,963,506 (-2,317,092, -1,601,714)	-1,977,735 (-2,338,882, -1,600,879)
Pancreatic	1,764,802 (1,430,319, 2,097,776)	0 (0, 0)	Dominated	-1,764,802 (-2,097,776, -1,430,319)	-1,764,802 (-2,097,776, -1,430,319)
Neuroendocrine	931,815 (751,850, 1,120,397)	0 (0, 0)	Dominated	-931,815 (-1,120,397, -751,850)	-931,815 (-1,120,397, -751,850)
Other	501,754 (409,813, 596,727)	0 (0, 0)	Dominated	-501,754 (-596,727, -409,813)	-501,754 (-596,727, -409,813)

CI: confidence interval; ICER: incremental cost-effectives ratio; INMB: incremental net monetary benefit; MASC: mammary analogue secretory carcinoma; N/A: not applicable; NSCLC: non-small cell lung cancer; QALY: quality-adjusted life year; USD: United States Dollar (2021).

Table 10: **Mean Tumor-agnostic and Tumor-specific Probabilistic Cost-effectiveness Outputs with Full Entrectinib Extrapolation, Including NGS Costs**

	Incremental cost, CAD Mean (95% CI)	Incremental QALYs Mean (95% CI)	ICER, CAD/QALY Mean (95% CI)	% Dominated	INMB at CAD 50,000/QALY, CAD Mean (95% CI)	INMB at CAD 100,000/QALY, CAD Mean (95% CI)	% CE at CAD 50,000/ 100,000
Tumor-agnostic	2,440,447 (2,011,306, 2,903,104)	0.766 (-0.141, 1.682)	6,029,951 (1,446,910, 25,232,641)	5	-2,402,125 (-2,856,405, -1,984,845)	-2,363,804 (-2,814,809, -1,934,694)	0/0
MASC	548,265 (311,918, 844,400)	4.364 (2.165, 6.202)	131,761 (75,259, 222,751)	0	-330,043 (-587,457, -118,469)	-111,821 (-365,381, 127,200)	0/0
NSCLC	5,305,633 (4,322,319, 6,312,296)	1.77 (-0.165, 4.115)	6,238,709 (1,305,932, 27,422,098)	4.1	-5,217,121 (-6,211,239, -4,238,024)	-5,128,609 (-6,134,622, -4,144,353)	0/0
Colorectal	3,512,879 (2,825,436, 4,236,672)	1.299 (-1.089, 3.568)	5,700,126 (954,174, 31,480,197)	18	-3,447,906 (-4,114,845, -2,811,365)	-3,382,934 (-4,030,853, -2,760,360)	0/0
Breast	8,592,952 (6,992,475, 10,234,159)	1.122 (-1.464, 3.324)	15,117,442 (2,510,510, 65,548,411)	19.9	-8,536,850 (-10,143,498, -6,930,395)	-8,480,748 (-10,117,038, -6,870,406)	0/0
Sarcoma	1,987,510 (1,590,431, 2,357,032)	0.149 (-1.687, 1.773)	11,446,754 (1,081,595, 60,019,483)	42.4	-1,980,056 (-2,333,277, -1,604,368)	-1,972,603 (-2,341,594, -1,582,228)	0/0
Thyroid	2,476,469 (1,976,093, 2,957,682)	-0.498 (-3.11, 2.232)	Dominated	65.1	-2,501,354 (-2,962,442, -2,025,393)	-2,526,239 (-3,021,544, -2,021,745)	0/0
Pancreatic	2,212,179 (1,792,905, 2,629,562)	0 (0, 0)	Dominated	N/A	-2,212,179 (-2,629,562, -1,792,905)	-2,212,179 (-2,629,562, -1,792,905)	N/A
Neuroendocrine	1,168,030 (942,444, 1,404,418)	0 (0, 0)	Dominated	N/A	-1,168,030 (-1,404,418, -942,444)	-1,168,030 (-1,404,418, -942,444)	N/A
Other	628,949 (513,701, 747,997)	0 (0, 0)	Dominated	N/A	-628,949 (-747,997, -513,701)	-628,949 (-747,997, -513,701)	N/A

CAD: 2021 Canadian dollars; CE; cost effective; CI: confidence interval; ICER: incremental cost-effectives ratio; MASC: mammary analogue secretory carcinoma; N/A: not applicable; NSCLC: non-small cell lung cancer; QALY: quality-adjusted life year.

Table 11: Mean Tumor-agnostic and Tumor-specific Probabilistic Cost-effectiveness Outputs with Full Entrectinib Extrapolation, Including NGS Costs and in USD

	Incremental cost, USD Mean (95% CI)	Incremental QALYs Mean (95% CI)	ICER, USD/QALY Mean (95% CI)	INMB at CAD 50,000/QALY, USD Mean (95% CI)	INMB at CAD 100,000/QALY, USD Mean (95% CI)
Tumor-agnostic	1,946,906 (1,604,552, 2,315,998)	0.766 (-0.141, 1.682)	4,810,491 (1,154,296, 20,129,750)	-1,916,334 (-2,278,744, -1,583,442)	-1,885,763 (-2,245,560, -1,543,434)
MASC	437,387 (248,838, 673,634)	4.364 (2.165, 6.202)	105,114 (60,039, 177,703)	-263,297 (-468,653, -94,511)	-89,207 (-291,489, 101,476)
NSCLC	4,232,655 (3,448,200, 5,035,737)	1.77 (-0.165, 4.115)	4,977,032 (1,041,828, 21,876,424)	-4,162,043 (-4,955,117, -3,380,953)	-4,091,431 (-4,893,994, -3,306,225)
Colorectal	2,802,456 (2,254,037, 3,379,874)	1.299 (-1.089, 3.568)	4,547,368 (761,208, 25,113,839)	-2,750,623 (-3,282,684, -2,242,812)	-2,698,791 (-3,215,679, -2,202,122)
Breast	6,855,167 (5,578,361, 8,164,467)	1.122 (-1.464, 3.324)	12,060,185 (2,002,800, 52,292,310)	-6,810,411 (-8,092,140, -5,528,835)	-6,765,655 (-8,071,032, -5,480,978)
Sarcoma	1,585,568 (1,268,792, 1,880,361)	0.149 (-1.687, 1.773)	9,131,834 (862,860, 47,881,518)	-1,579,622 (-1,861,410, -1,279,911)	-1,573,676 (-1,868,045, -1,262,248)
Thyroid	1,975,643 (1,576,460, 2,359,539)	-0.498 (-3.11, 2.232)	Dominated	-1,995,496 (-2,363,336, -1,615,790)	-2,015,348 (-2,410,486, -1,612,880)
Pancreatic	1,764,802 (1,430,319, 2,097,776)	0 (0, 0)	Dominated	-1,764,802 (-2,097,776, -1,430,319)	-1,764,802 (-2,097,776, -1,430,319)
Neuroendocrine	931,815 (751,850, 1,120,397)	0 (0, 0)	Dominated	-931,815 (-1,120,397, -751,850)	-931,815 (-1,120,397, -751,850)
Other	501,754 (409,813, 596,727)	0 (0, 0)	Dominated	-501,754 (-596,727, -409,813)	-501,754 (-596,727, -409,813)

CI: confidence interval; ICER: incremental cost-effectives ratio; INMB: incremental net monetary benefit; MASC: mammary analogue secretory carcinoma; N/A: not applicable; NSCLC: non-small cell lung cancer; QALY: quality-adjusted life year; USD: United States Dollar (2021).

Table 12*:* **Mean Tumor-agnostic and Tumor-specific Probabilistic Cost-effectiveness Outcomes with Restricted Effectiveness of Entrectinib and Adjusted NICE Costs**

	Incremental cost, CAD[a] Mean (95% CI)	Incremental QALYs Mean (95% CI)	ICER[a], CAD/QALY Mean (95% CI)	% Domi-nated	INMB at CAD 50,000/QALY, CAD[a] Mean (95% CI)	% CE at CAD 50,000/ QALY[a]	INMB at CAD 100,000/QALY, CAD[a] Mean (95% CI)	% CE at CAD 100,000/ QALY[a]
Tumor-agnostic	68,688 (35,972, 92,044)	0.126 (-0.419, 0.421)	871,617 (180,608, 4,598,058)	20.5	-62,364 (-81,959, -40,869)	0.1	-56,040 (-89,485, -29,952)	0.1
MASC	203,858 (169,175, 250,311)	1.125 (0.634, 1.432)	190,796 (136,841, 318,641)	0	-147,620 (-194,637, -112,782)	0	-91,383 (-149,144, -49,201)	0
NSCLC	72,564 (-8,299, 123,784)	0.494 (-0.134, 0.873)	188,334 (3,382, 704,108)	3.7	-47,861 (-87,383, 15,893)	4.9	-23,159 (-73,922, 36,341)	16.1
Breast	86,399 (-12,297, 124,910)	0.173 (-1.12, 0.578)	1,347,506 (175,811, 2,678,828)	16	-77,769 (-107,241, -31,334)	3.7	-69,139 (-119,148, -39,389)	3.7
Colorectal	51,676 (3,590, 78,273)	0.037 (-0.437, 0.224)	1,526,017 (207,576, 5,525,576)	23.3	-49,810 (-75,292, -19,734)	1.9	-47,944 (-80,735, -17,189)	2.2
Sarcoma	78,834 (10,951, 114,198)	-0.008 (-1.064, 0.488)	Dominated	38.2	-79,228 (-111,042, -46,235)	1.9	-79,622 (-135,810, -37,959)	1.9
Thyroid	61,110 (-58,574, 110,808)	-0.357 (-2.592, 0.591)	Dominated	53.5	-78,947 (-120,863, -26,151)	10.1	-96,783 (-207,468, -30,155)	10.1
Pancreatic	23,743 (2,407, 63,559)	0 (0, 0)	Dominated	N/A	-23,743 (-63,559, -2,407)	N/A	-23,743 (-63,559, -2,407)	N/A
Neuro-endocrine	139,054 (41,913, 273,642)	0 (0, 0)	Dominated	N/A	-139,054 (-273,642, -41,913)	N/A	-139,054 (-273,642, -41,913)	N/A
Other	45,502 (8,949, 78,896)	0 (0, 0)	Dominated	N/A	-45,502 (-78,896, -8,949)	N/A	-45,502 (-78,896, -8,949)	N/A

CAD: 2021 Canadian dollars; CE; cost effective; CI: confidence interval; ICER: incremental cost-effectives ratio; MASC: mammary analogue secretory carcinoma; N/A: not applicable; NSCLC: non-small cell lung cancer; QALY: quality-adjusted life year.

Figure 1: Partitioned Survival Analysis Schematic.

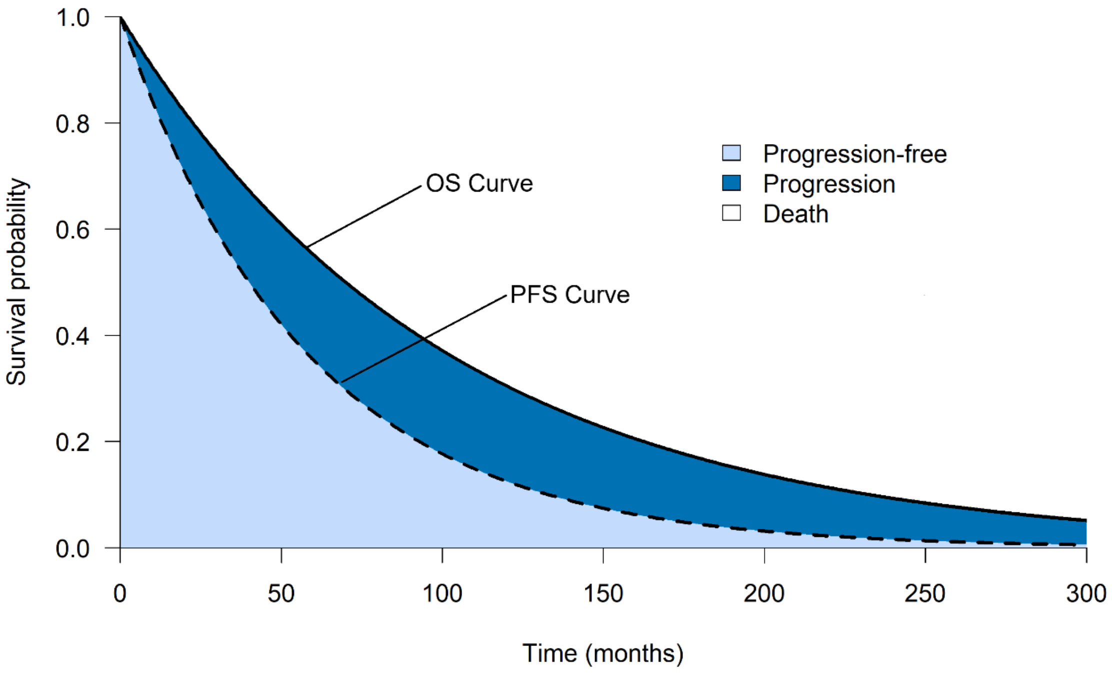

OS: Overall survival; PFS: Progression-free survival

Figure 2: Tumor-Specific Cost-effectiveness Plane, Without NGS Costs

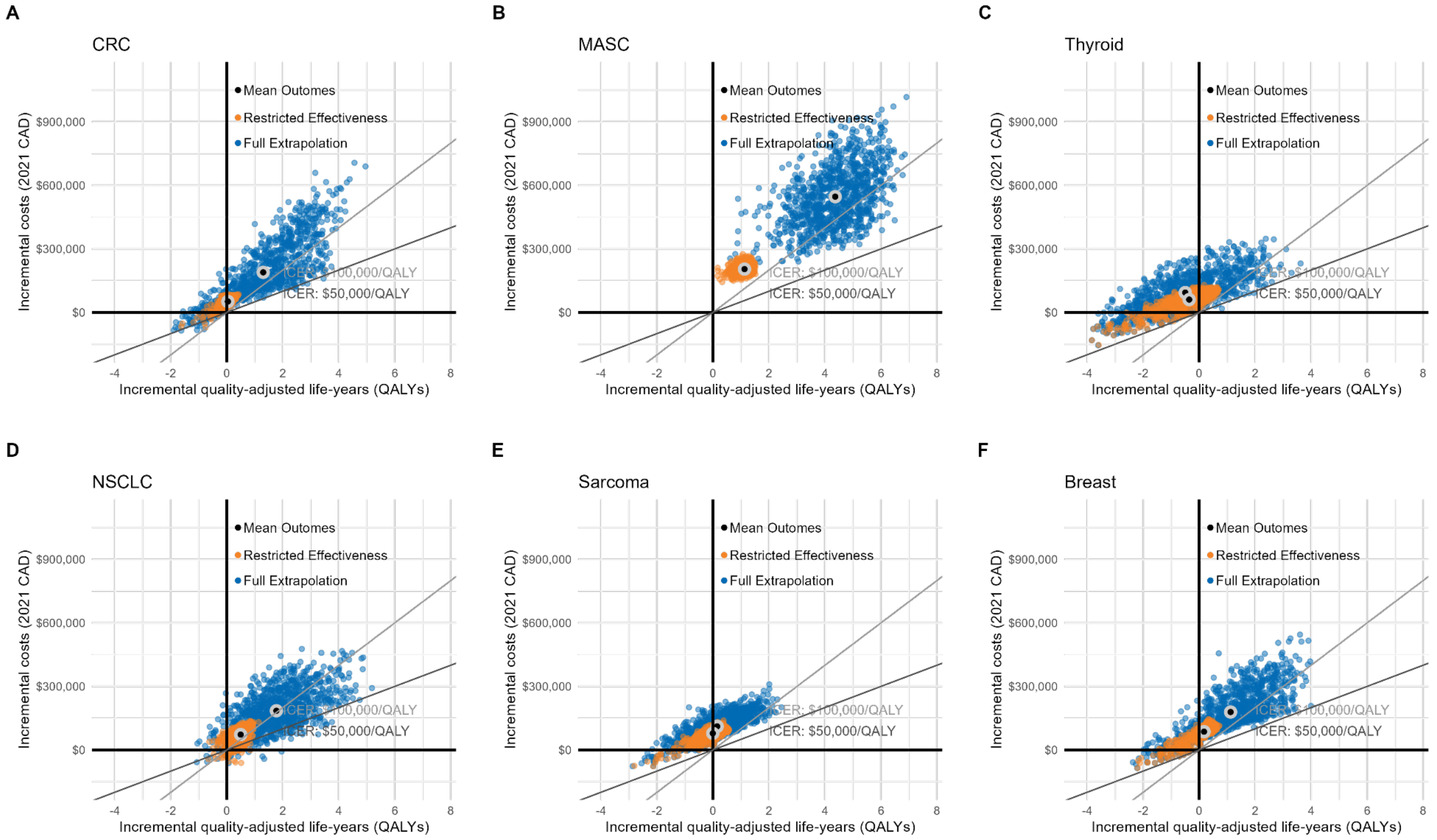

Tumor indications not included: pancreatic, neuroendocrine, and other. Entrectinib survival is equivalent to standard care for these indications, and so only incremental costs are accrued. CAD: 2021 Canadian dollars; CRC: colorectal; ICER: Incremental cost-effectiveness ratio; MASC: Mammary-analogue secretory carcinoma; NSCLC: Non-small cell lung cancer; QALY: Quality-adjusted life years; WTP: Willingness-to-pay.

Figure 3: Tumor-agnostic* Cost Effectiveness Plane with Restricted Effectiveness of Entrectinib, Including NGS Costs.

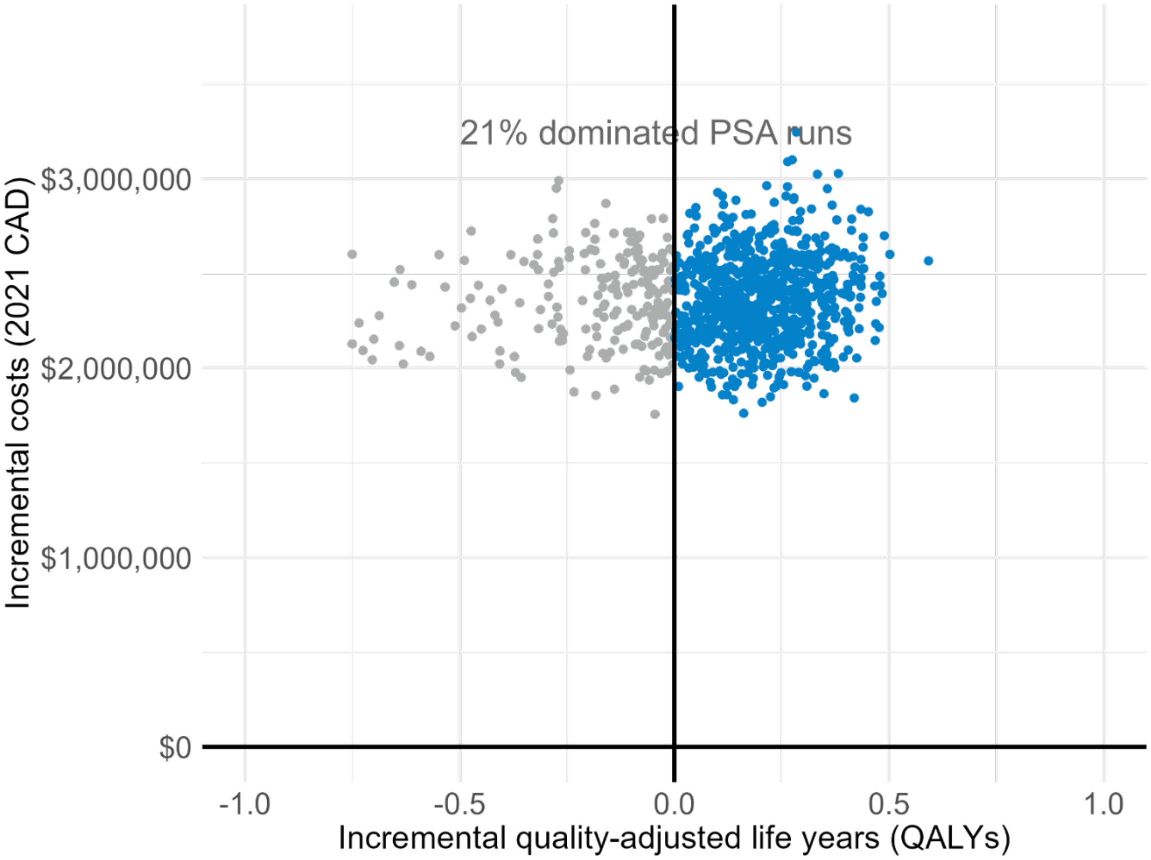

* Separate outcomes for each tumor indication are weighted using cancer prevalence estimates. CAD: Canadian dollars; ICER: incremental cost-effectiveness ratio.

Figure 4: Tumour-agnostic* Expected Value of Perfect Information

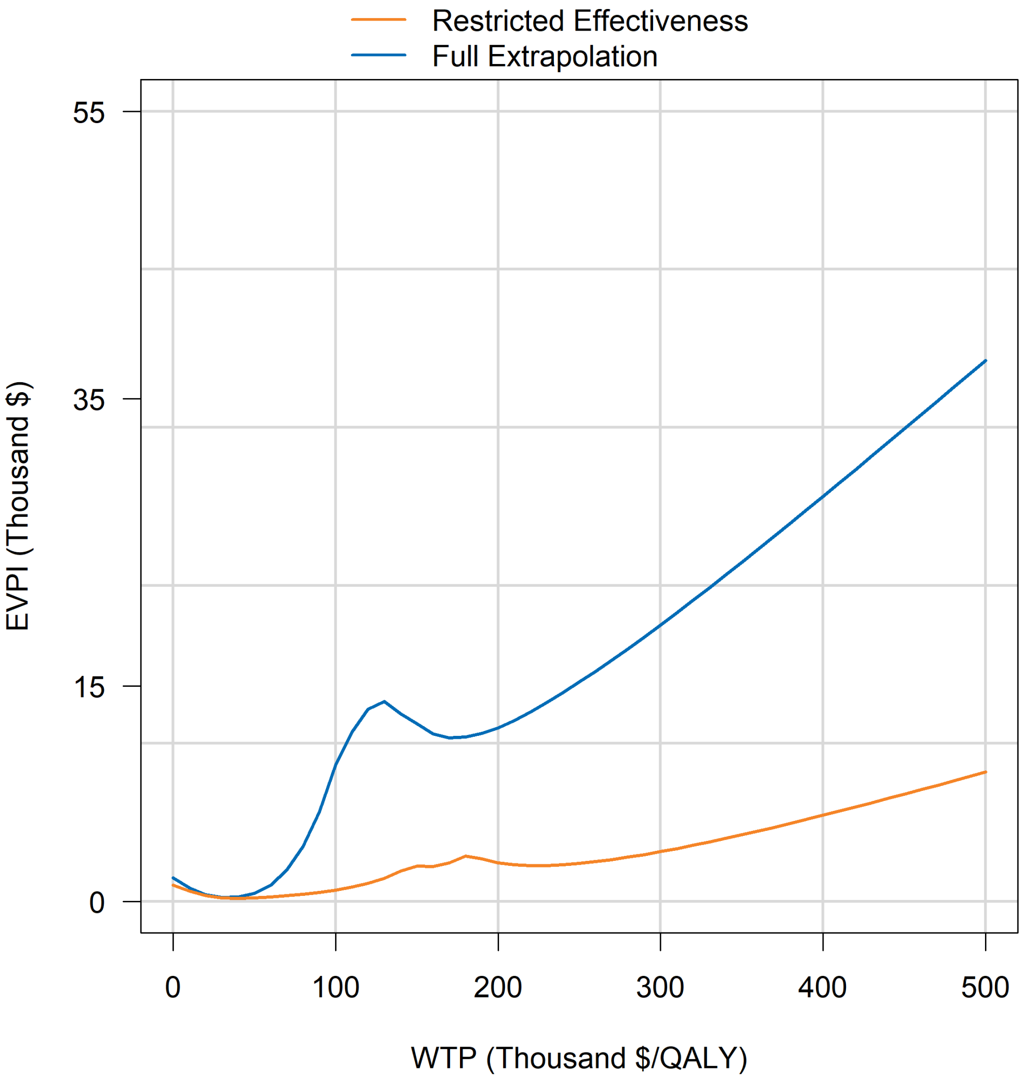

* Separate outcomes for each tumor indication are weighted using cancer prevalence estimates. Results presented in 2021 Canadian dollars. EVPI: Expected value of perfect information; QALY: Quality-adjusted life years; WTP: Willingness-to-pay.

Figure 5: Tumour-specific Expected Value of Perfect Information

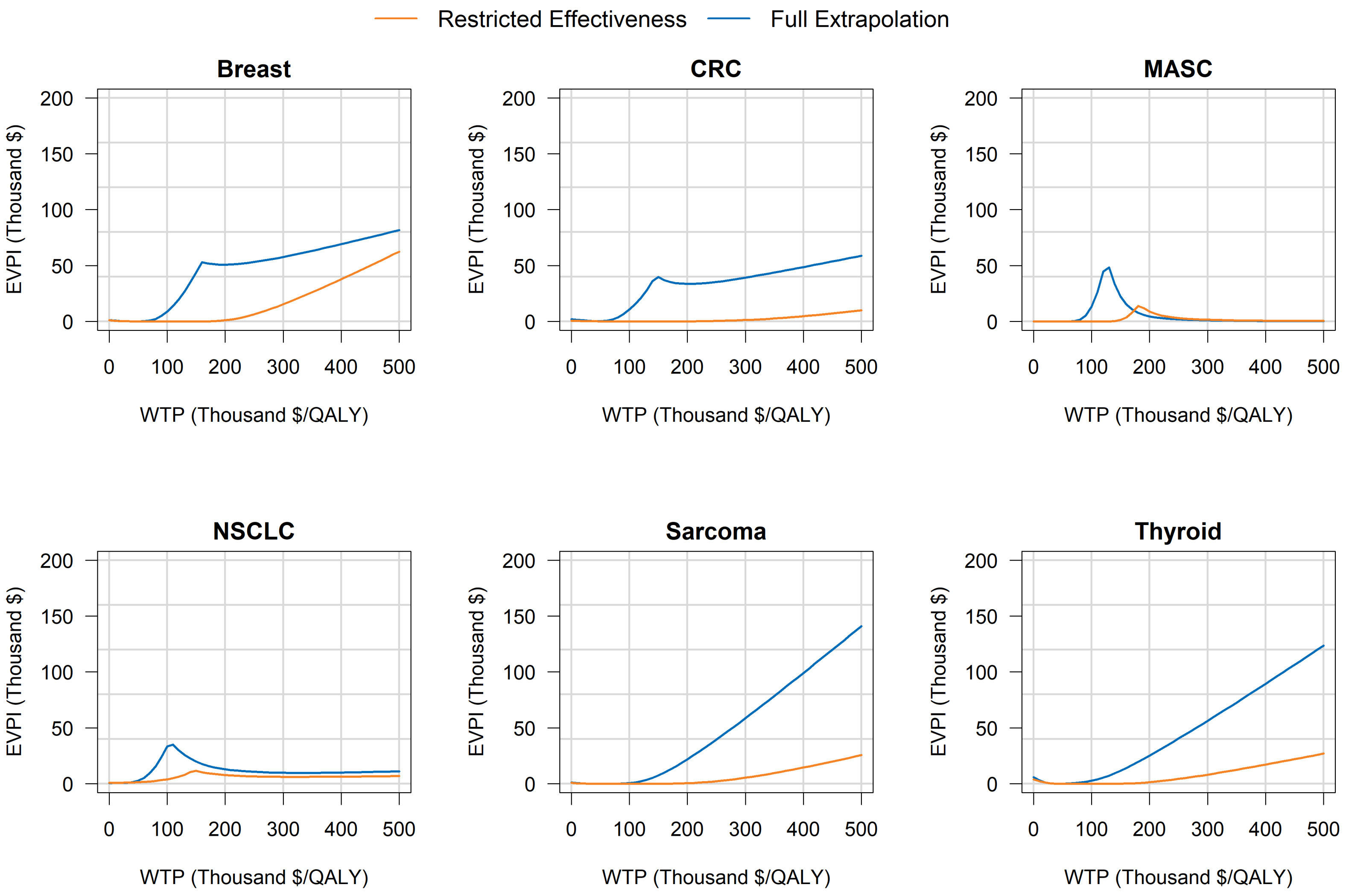

Tumor indications not included: pancreatic, neuroendocrine, and other. Entrectinib survival is equivalent to standard care for these indications, and so the EVPI is zero. Results presented in 2021 Canadian dollars. CRC: colorectal; EVPI: Expected value of perfect information; MASC: Mammary-analogue secretory carcinoma; NSCLC: Non-small cell lung cancer; QALY: Quality-adjusted life years; WTP: Willingness-to-pay.

References

1. Jalal, H., et al. *An overview of R in health decision sciences*. Medical decision making, 2017. **37**, 735-746 DOI: https://doi.org/10.1177/0272989x16686559.
2. Bank of Canada. *[Internet] Annual Exchange Rates*. 2024/06/05]; Available from: https://www.bankofcanada.ca/rates/exchange/annual-average-exchange-rates/.
3. Statistics Canada. *Table 18-10-0004-08 Consumer Price Index, monthly, percentage change, not seasonally adjusted, Canada, provinces, Whitehorse and Yellowknife — Health and personal care*. 2024 2024/06/11].
4. CADTH, *Entrectinib (Rozlytrek) For the Treatment of Extracranial Solid Tumours with NTRK gene fusion*. 2023: CADTH Reimbursement Review.
5. Nagy, B., et al. *Lessons learned from the application of the HEcoPerMed guidance to three modeling case studies*. Personalized Medicine, 2023. **20**, 401-411 DOI: https://doi.org/10.2217/pme-2023-0040.
6. Rutten-van Mölken, M., et al. *HEcoPerMed, personalized medicine from a health economic perspective: lessons learned and potential opportunities ahead*. 2023. **20**, 299-303 DOI: https://doi.org/10.2217/pme-2022-0074.
7. Vellekoop, H., et al. *Guidance for the harmonisation and improvement of economic evaluations of personalised medicine*. Pharmacoeconomics, 2021. **39**, 771-788 DOI: https://doi.org/10.1007/s40273-021-01010-z.
8. Huygens, S., et al. *Cost-effectiveness analysis of treating patients with NTRK-positive cancer with the histology-independent therapy entrectinib*. Value in Health, 2023. **26**, 193-203 DOI: https://doi.org/10.1016/j.jval.2022.08.006.
9. Coyle, D., A. Haines, and K. Lee *The development of a model validation tool to assist in the conduct of economic evaluations*. Canadian Journal of Health Technologies, 2024. **4**, DOI: https://doi.org/10.51731/cjht.2024.862.
